# An identification of invariants in life history traits of amphibians and reptiles

**DOI:** 10.1002/ece3.5978

**Published:** 2020-01-08

**Authors:** Konstantin Hallmann, Eva Maria Griebeler

**Affiliations:** ^1^ Institute of Organismic and Molecular Evolution – Evolutionary Ecology Johannes Gutenberg‐University Mainz Mainz Germany

**Keywords:** allometric regressions, Anura, Caudata, invariance, isometric variation, reproductive traits

## Abstract

While many morphological, physiological, and ecological characteristics of organisms scale with body size, some do not change under size transformation. They are called invariant. A recent study recommended five criteria for identifying invariant traits. These are based on that a trait exhibits a unimodal central tendency and varies over a limited range with body mass (type I), or that it does not vary systematically with body mass (type II). We methodologically improved these criteria and then applied them to life history traits of amphibians, Anura, Caudata (eleven traits), and reptiles (eight traits). The numbers of invariant traits identified by criteria differed across amphibian orders and between amphibians and reptiles. Reproductive output (maximum number of reproductive events per year), incubation time, length of larval period, and metamorphosis size were type I and II invariant across amphibians. In both amphibian orders, reproductive output and metamorphosis size were type I and II invariant. In Anura, incubation time and length of larval period and in Caudata, incubation time were further type II invariant. In reptiles, however, only number of clutches per year was invariant (type II). All these differences could reflect that in reptiles body size and in amphibians, Anura, and Caudata metamorphosis (neotenic species go not through it) and the trend toward independence of egg and larval development from water additionally constrained life history evolution. We further demonstrate that all invariance criteria worked for amphibian and reptilian life history traits, although we corroborated some known and identified new limitations to their application.

## Graphical abstract

While many morphological, physiological, and ecological characteristics of organisms scale with body size, some do not change under body size transformation and they are called invariant. An invariant trait exhibits a unimodal central tendency and varies over a limited range with body mass (type I), or it does not vary systematically with body mass (type II). We methodologically improved these criteria and applied them to life history traits of amphibians, Anura, Caudata (ten traits), and reptiles (eight traits). The numbers of invariant traits identified by different criteria differed across amphibian orders and between amphibians and reptiles.

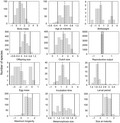

## 1 INTRODUCTION

Allometry (biological scaling) describes the dependence of a biological variable on an organism's body size. Virtually, all morphological, physiological, and ecological characteristics including life history traits of organisms seem to vary predictably with their body size (e.g., Blueweiss et al., 1978; Brown, Gillooly, Allen, Savage, & West, 2004; Peters, 1983; Schmidt‐Nielsen, 1984). The relationships between these characteristics (C) and body size (S) are typically expressed as a power function (C = *a* S*^b^*) with the scaling exponent *b* and normalization constant *a*. Then, a linear scaling model results from a log–log transformation of the power function. Here, the scaling exponent *b* is the slope and log_10_(*a*) is the intercept of this straight line. For several decades, biologists have been investigating the mechanistic processes underlying allometric relationships (e.g., Brown et al., 2004; Kooijman, 1993; West, Brown, & Enquist, 1997).

In the field of life history evolution, two classical but contrasting thinking exist. The first sees variation in traits due to their allometric scaling to body size, whereas for the second a life history is shaped by natural selection acting fairly independently of body size (Charnov, 1993). Numerous allometric regressions with a strong focus on vertebrates are in the literature (e.g., Blueweiss et al., 1978; Brown et al., 2004; Dol'nik, 2000; Hallmann & Griebeler, 2018; Hendriks & Mulder, 2008; Peters, 1983; Schmidt‐Nielsen, 1984). They successfully relate species traits to body mass and thus provide support for the first evolutionary hypothesis. Much fewer studies, however, report so‐called invariant traits (Beverton & Holt, 1959; Charnov, 1993) providing evidence for the second.

Beverton and Holt (1959) were the first who identified such invariant traits, that is, between traits of growth and mortality across fish species and populations (Beverton, 1963). These authors described invariants by products or ratios of two traits (dimensionless numbers). Beverton–Holt invariants were later demonstrated for other pairs of traits (e.g., Charnov, 1993) and also for other vertebrate groups including squamate reptiles (lizards and snakes; Shine & Charnov, 1992). Charnov (1993) formulated his general concept of invariance that some attributes of an object of interest remain unchanged under a specific transformation. Body size transformation is only one of these and links the concept of invariance to that of allometric scaling.

Savage, White, Moses, Ernest, and Enquist (2006) were the first who brought significant clarification into the concept of invariance. They clearly elaborated the two different types of invariance under body size transformation addressed by Beverton and Holt (1959) and Charnov (1993). In type I invariance, a life history trait exhibits a unimodal central tendency and varies over a limited range. This type refers to that a trait shows virtually no variability. In type II invariance, a life history trait does not vary systematically with body size. Traits conforming to type I or II invariance will not scale allometrically with body size (Savage et al., 2006). Such traits provide evidence against the generality of allometric scaling in biology (scaling laws) and especially that body size is a main driver of the evolution of life history traits. Savage et al. (2006) also noted that traits could conform to both types of invariance (hereafter type I + II invariance).

The concept of invariance is still discussed in the literature (Mangel, 2017; Morrow, Ernest, & Kerkhoff, 2019; Price et al., 2014; Thorson, Munch, Cope, & Gao, 2017). This is not only due to its ecological and evolutionary implications but also due to methodological problems with respect to the identification of invariant traits. For example, two traits scaling with identical exponents to body size may be, or may not, be invariant when regressed against each other. Further, an illusion of invariance can arise, when one trait is a fraction of another (Nee, Colegrave, West, & Grafen, 2005). Usually, the mechanistic processes causing trait invariance are unknown (Charnov, 1993) and only statistical or probabilistic evidence exists. The latter is in particular problematic because we need a threshold (such as the significance level of a test) which a trait should pass before being considered invariant (Charnov, 1993). This all led to the questioning of the whole concept of invariance by several authors (for a discussion on this topic see Günther & Morgado, 2005; Nee et al., 2005; Nespolo, 2005; Savage et al., 2006), but also to an improvement of methods available for the identification of invariant traits (Price et al., 2014).

Price et al. (2014) elaborated an objective statistical framework on type I, type II, and type I + II invariance (Savage et al., 2006). These authors recommended five different criteria for identifying invariant traits or, more precisely, traits approaching invariance under body size transformation. Their criteria of which two refer to type I invariance (1 and 2) and three to type II invariance (3 through 5) are as follows: (1) a low variance in the trait compared to that seen in body size (type I). (2) Unimodal or normal distribution of trait values suggesting that an optimum value exists across the taxon under study (type I). (3) Either a low coefficient of determination (*R^2^* value of the ordinary least squares [OLS] regression line, log–log plot, body size has a low explanatory power for the trait of interest, residual variation is large, type II), or (4) a low slope, when regressing the trait against body size (log–log plot, OLS regression, size has a low explanatory power for the trait of interest, type II). And finally (5) an isometric relationship (slope = 1) between two arbitrary traits (their ratio is invariant across the spectrum of variation in the two traits and thus may also be invariant when regressed against body size, log–log plot, type II). For criterion (1), the authors additionally noted that all biological quantities are variable and that hence the variability seen in a life history trait under study should at least be much smaller than that seen in body size (Price et al., 2014). They also mentioned that these five criteria are not mutually exclusive in terms of identification of invariances as statistical interrelations exist (Savage et al., 2006).

In this paper, we chose amphibians and reptiles to disentangle the relationship between their life history strategies and body size by identifying invariant life history traits. We further address methodological limitations of the Price et al. (2014) framework by studying invariant traits for both vertebrate groups. Previous studies have shown that amphibians and reptiles differ considerably in their ranges of body mass (Collar, Schulte, & Losos, 2011) and in the variability seen in their life history strategies (in terms of trait ranges and existing combinations of trait values, Duellman & Trueb, 1994; Morrow et al., 2019). Although there were larger forms in the Paleozoic, today's amphibians are generally small and body size variability is small when compared to that seen in any other vertebrate taxon. The largest salamander has a total length (TL) of about 1,500 mm, and the TL of the largest frog is about 300 mm (Oliveira, São‐Pedro, Santos‐Barrera, Penone, & Costa, 2017). Amphibians and especially salamanders show the highest diversity in life history strategies across vertebrates (Morrow et al., 2019), and an amazing diversity exists in amphibian reproductive modes. Amphibians show oviparity (yolk supports embryonic development until eggs hatch), ovoviviparity (lecithotrophy), and viviparity (matrotrophy) (Duellman & Trueb, 1994; Haddad & Prado, 2005; Lion et al., 2019; Wells, 2007). Although an aquatic reproduction is ancestral, a terrestrial reproduction is also seen in amphibians. Many species have evolved ways of depositing terrestrial eggs, but still retain the aquatic larval/tadpole stage. Others are completely independent of water by eliminating the free‐swimming larva/tadpole. They retain their young on or within the body (inside or outside the reproductive tract) until development is complete or produce direct‐developing eggs with large yolk reserves from which juveniles (miniature adults) hatch. Compared to amphibians, extremely large body sizes evolved in reptiles (the largest extant lizard species is the Komodo dragon *Varanus komodoensis*; Collar et al., 2011) and Collar et al. (2011) pointed out that monitor lizards even show the largest size range seen within any genus of vertebrates. For reptiles, many studies on allometric relationships of biological traits including life history traits exist (see Dol'nik, 2000; Dunham & Miles, 1985; Fitch, 1970; Hallmann & Griebeler, 2015, 2018; Peters, 1983; Scharf et al., 2014; Schmidt‐Nielsen, 1984), whereas such on amphibians are comparatively rare (e.g., Dol'nik, 2000; Duellman & Trueb, 1994; Earl & Whitman, 2015). All this indicates that size matters much in reptiles, but less in amphibians, making both vertebrate groups good models for studying invariance of life history traits toward body size.

We examined whether eleven life history traits of the amphibians, and the two orders Anura, and Caudata (traits cover the egg, larval, and adult stage; a metamorphosis at the end of the larval stage is seen in the majority of amphibians, exceptions are neotenic species that mature in the larval stage, Lynn, 1961; sample sizes on traits of Gymnophiona were too small for analyzing invariances of traits, Figure S1) and eight life history traits of the reptiles approach invariance. As Hallmann and Griebeler (2018) showed that allometric relationships on life history traits and body mass do not differ between the reptilian clades Crocodilia, Squamata, and Testudines, and thus, traits are not type II invariant in these taxonomic groups, we did not consider these reptile subgroups in our study. In addition, the majority of species covered in our reptile dataset are squamates (*n* = 294), and the sample size of turtles (*n* = 52) and of crocodiles (*n* = 22) is about one magnitude smaller than that on squamates (Hallmann & Griebeler, 2018). Note that overall sample sizes on individual traits of reptile species (except for body mass) are smaller than the number of species covered by this database. When the analysis is limited to turtles and crocodiles, trait samples will shrink even more, and this questions the validity of any result obtained on both reptilian subgroups.

To study trait invariances in the amphibians and reptiles, we first methodologically improved and then applied the five criteria referring to type I and II invariance, respectively, being elaborated in Price et al. (2014). Due to the above‐mentioned differences in life history trait variability and body size ranges known between both vertebrate classes, we expected a higher frequency of invariant life history traits in amphibians (Anura, Caudata) than in reptiles. This could indicate that in amphibians, life history evolution is more constrained by egg and larval/tadpole development (in aquatic and/or terrestrial environments) and less shaped by adult size, whereas size is a very important evolutionary factor for reptiles with a less complex life cycle.

In amphibians, not only are studies on invariant traits rather rare in the literature, but also such on allometric relationships (without and with phylogenetic correction) relating life history traits to body mass (e.g., Dol'nik, 2000; Duellman & Trueb, 1994; Earl & Whitman, 2015). In our study, we further establish such allometric relationships on life history traits and differences in relationships of life history traits seen between amphibians and reptiles. Having regressions linking life history traits to body size in amphibians is not only important for other comparative studies on extant taxa. In paleobiology, such regressions are routinely used as comparative models for extinct vertebrates (e.g., Werner & Griebeler, 2011, 2013).

Finally yet importantly, as Price et al. (2014) provided several criteria on type I and type II invariance, respectively, and we made some methodological improvements on their criteria, we use our analysis on species groups to identify their methodological limitations in identifying invariant traits.

## 2 MATERIAL AND METHODS

### 2.1 Data collection

Amphibians. The basis of our amphibian dataset (Hallmann & Griebeler, 2019) was the comprehensive database on life history traits of European amphibians (86 amphibian species of which 50 species are Anura and 36 are Caudata) from Trochet et al. (2014) and the AmphiBIO database (Oliveira et al., 2017), which covers amphibians from all over the world (*n* > 6,500). As a measure of animal size, we chose species’ adult body mass (g) instead of body length or snout–vent–length also being listed in both databases, because Anura and Caudata considerably differ in shapes.

Besides adult body mass, we extracted the following life history traits of species from the Trochet et al. (2014) database. These were egg mass (g), clutch size (this term is used herein for both the number of eggs and also for the number of offspring in viviparous species), metamorphosis size (mm), and age at (sexual) maturity (days needed for becoming sexually mature, this term is also used for the amphibians not going through metamorphosis and maturing within the larval stage, e.g., the Mexican Axolotl, *Ambystoma mexicanum*, or the olm, *Proteus anguinus*, Lynn, 1961; Safi et al., 2004; Voss, Epperlein, & Tanaka, 2009). From the AmphiBIO dataset, we extracted the five life history traits maximum longevity (max. longevity, years), minimum and maximum age at (sexual) maturity (years), minimum and maximum size at (sexual) maturity (mm), minimum and maximum litter (clutch) size, and minimum and maximum offspring (or egg) size (mm). For complementing the Trochet et al. (2014) database with AmphiBIO records on species’ age and size at maturity, offspring size, and clutch size, we averaged the respective minimum and maximum values listed in AmphiBIO. Units of traits covered in both databases were standardized to that used by Trochet et al. (2014).

To cover more traits from the egg and larval phase in an amphibian's life, we added information on the length of the incubation time of the embryo (incubation time, days), the length of the larval/tadpole phase (larval period, days), and birthweight (g) to each of the species records. For these traits, but also to increase information on the other traits of species, we compiled the multivolume encyclopedia on European amphibians (Bischoff, 1998; Böhme, 1981, 1984, 1986, 1993, 1999; Joger & Stümpel, 2005), the Internet database AnAge (Tacutu et al., 2013), primary literature, field guides, and textbooks. For the complete and detailed list of all references used beyond those consulted by Trochet et al. (2014) and Oliveira et al. (2017), please refer to Hallmann and Griebeler (2019). To ensure sufficient data quality, we did not consider anecdotal remarks (without any reference) given in the aforementioned sources. In cases where we found ranges or multiple values for species traits, we always averaged these for statistical analysis.

**Figure 1:**
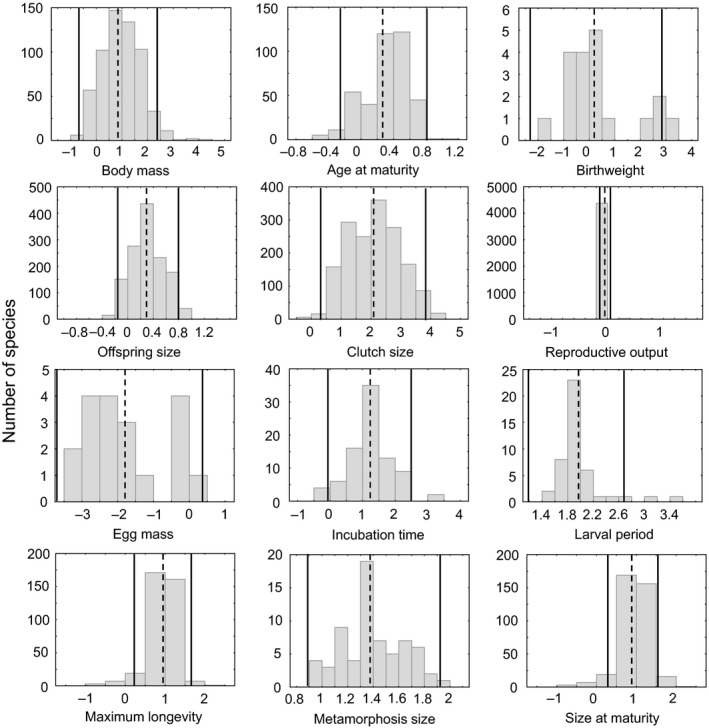
Histograms on log_10_‐transformed data for each of the amphibian life history traits studied (criteria 1 and 2, type I invariance). In all plots, the dashed line represents the mean value of each distribution and the two solid lines represent plus/minus two standard deviations (±2 *SD*) of the mean. N_body mass_ = 597, N_age at maturity_ = 399, N_birthweight_ = 19, N_offspring size_ = 1,330, N_clutch size_ = 1629, N_reproductive output_ = 4,432, N_egg mass_ = 19, N_incubation time_ = 85, N_larval period_ = 44, N_maximum longevity_ = 369, N_metamorphosis size_ = 67, N_size at maturity_ = 371. For histograms and sample sizes on life history traits of Anura, Caudata, and Gymnophiona, see Figure S1

Our final amphibian database (Hallmann & Griebeler, 2019) covers a total of 6,779 species of which 5,974 are Anura, 619 are Caudata, and 185 are Gymnophiona. It provides information on species’ maximum adult body mass (grams, overall *n* = 597) and on eleven other life history traits covering its life. These are age (years, *n* = 399) and size at (sexual) maturity (years, *n* = 371), egg mass (g, *n* = 19), birthweight (g, *n* = 19), offspring size (mm, *n* = 1,333), clutch (litter) size (*n* = 1,629), reproductive output (maximum number of reproduction events per year, *n* = 4,435), incubation time (days, *n* = 85), larval period (days, *n* = 44), metamorphosis size (mm, *n* = 67), and maximum longevity (years, *n* = 369). Histograms and sample sizes on trait values are in Figure 1 for all amphibian species and in Figure S1 for Anura, Caudata, and Gymnophiona, separately. Size at birth is assessed twice in our database as offspring size (mm) and birthweight (g). Offspring size ignores differences in shapes of amphibian species, and our reptile database (Hallmann & Griebeler, 2018) provides birthweight (g). Our amphibian database assesses clutch frequency as a species’ maximum number of reproductive events per year (reproductive output), whereas the trait numbers of clutches per year in the reptile database refer to the average number of clutches per year seen in a species (Hallmann & Griebeler, 2018).

Reptiles. All reptile life history data (*n* = 369) were taken from Hallmann and Griebeler (2018). From this global dataset, we used in this study the eight life history traits egg weight (egg mass, g, *n* = 62), clutch size (*n* = 210), (number of) clutches p.a. (= per annum, *n* = 137), incubation time (days, *n* = 174), birthweight (g, *n* = 78), age at (female sexual) maturity (days, *n* = 121), size at (sexual) maturity (cm, *n* = 54), and maximum longevity (years, *n* = 280). The amphibian traits metamorphosis size and larval period are apparently not applicable to reptiles. In reptiles, birth size is only assessed by birthweight (and not by offspring size).

### 2.2 Data analysis

All statistical analyses were performed with the statistical software R v3.5.1 (R Development Core Team, 2018) and additional packages (see below) available for this software.

Identifying life history invariants. We basically followed the approach given in Price et al. (2014) but made some improvements to their statistical framework. We applied the five criteria given by these authors on life history invariances to all amphibians, to each of the amphibian orders the Anura and Caudata (sample sizes on Gymnophiona were too small, Figure S1; for our few results on Gymnophiona, see Table S1), and to all reptiles.

Criterion (1)—low variance in the life history trait compared to that seen in body mass (type I). Contrary to Price et al. (2014) and in order to apply criterion (1) on type I invariance, we first normalized and transformed life history traits and body masses of amphibian and reptilian species. We aimed to account for the highly different ranges and medians of body masses seen in amphibians and reptiles. For normalization, we calculated the median for each life history trait (raw data) and body mass (raw data) for the respective group under study (all amphibians, orders Anura and Caudata, reptiles). To retain the relative variance of the traits, we next divided the raw data by the respective median. We then log_10_‐transformed normalized data to ensure that data were approximately evenly distributed (Steuer, Morgenthal, Weckwerth, & Selbig, 2007). From these new datasets, we finally calculated the ratio between the body mass variance and the trait variance (“body mass var/trait var”) for each life history trait. We assumed that a trait fulfilled criterion (1) when “body mass var/trait var” was larger than unity.

Criterion (2)—unimodal distribution with 95% of observations seen within the plus/minus two standard deviation range of the mean (type I). This criterion is based on that values of type I invariant traits basically follow a normal distribution (unimodality was only inspected by eye in Price et al., 2014). Before applying criterion (2) to each of the four species groups studied, we first log_10_‐transformed raw data of all amphibians and reptiles. We next performed Hartigan's dip tests to test for the unimodality of trait distributions using the function *dip.test* from the R‐package “diptest” (Maechler, 2015) for each species group. We then calculated as suggested by Price et al. (2014) the percentage of values that fell within plus/minus two standard deviations (±2 *SD*) of the mean for each group, which are expected to exceed 95% under a normal distribution.

Criterion (3)—low coefficient of determination (*R* ^2^) and criterion (4)—low slope when regressing life history traits against body mass (type II). Following the study of Price et al. (2014), we assumed that a coefficient of determination smaller than 0.05 (criterion 3) and that a nonsignificant slope (criterion 4) of the regression line indicates trait invariance. Prior to all regression analyses, we log_10_‐transformed values of life history traits and body masses of species. As in Price et al. (2014), we first established linear OLS regression models on each life history trait and for all species groups studied. We, therefore, applied the function *lm* from the R‐package *stats* (R Development Core Team, 2018) using the life history trait as the dependent variable and body mass as the independent variable. To assess criterion (4) and thereby correct for a shared evolutionary history of species (i.e., for potential errors in the estimated regression slopes and their significance), we additionally conducted phylogeny‐informed regression analysis. For amphibian groups, these phylogenetic generalized least squares (PGLS) regression models were derived from the function *gls* provided in the R‐package *nlme* (Pinheiro, Bates, DebRoy, Sarkar, & Team TRC, 2015). We applied the modified version of the Brownian motion model from Pagel (1999) as a trait evolution model and created the phylogenetic correlation structure with the function *corPagel* (Paradis, Claude, & Strimmer, 2004). Depending on the species sample under study, we generated the phylogenetic trees needed for PGLS by pruning a large‐scale phylogenetic tree on amphibian species (7,238 species, Jetz & Pyron, 2018). As this time‐calibrated tree is not ultrametric, we had to use weights for the *gls* function. We thus set the diagonal of the variance–covariance matrix to the fixed variance weights for the GLS model with the constructor‐function *varFixed* to correct for the noncontemporaneous tips within the phylogenetic tree of the amphibians (Revell, 2012). Then, we used the function *intervals* from the R‐package *nlme* to calculate the confidence intervals of estimated intercepts and slopes.

The allometric OLS and PGLS regression models on reptilian traits used in this study and their statistics are taken from Hallmann and Griebeler (2018). This paper used a composite topology for carrying out PGLS regression analysis. This topology was established from a phylogenetic tree of squamates (Pyron & Burbrink, 2014), of crocodiles (Oaks, 2011), and of turtles (Guillon, Guéry, Hulin, & Girondot, 2012).

Criterion (5)—isometric relation (i.e., the slope is unity in a log–log plot) between two life history traits (type II). Following Price et al. (2014), we established linear models for pairs of life history traits for all species groups using standard major axis (SMA) regression analysis on log_10_‐transformed data. Invariant traits have a slope of unity under SMA (Price et al., 2014). We checked this by inspecting whether unity is within the 95% confidence interval of the slope estimate. These regressions were established using the function *sma* of the R‐package “smatr” (Warton, Duursma, Falster, & Taskinen, 2012). With these regressions, we aimed to identify dimensionless numbers, which indicate trade‐offs between two life history traits (Beverton & Holt, 1959; Charnov, 1993).

## 3 RESULTS

The numbers of traits identified as invariant by different criteria differed across the four species groups studied (Table 1).

**Table 1:** A brief summary of results on criteria (1) through (4) on the invariance of life history traits in all amphibians, the amphibian orders Anura and Caudata, and in reptiles

	ASM	BW	OS	CS	RO/ CPA	EM	I	L	ML	MS	SSM
*All amphibians*											
Criterion 1 (variance ratio)	+	−	+	−	+	−	+	+	+	+	+
Criterion 2											
Unimodality	−	+	−	−	+	+	+	+	−	+	−
95% within ±2 *SD*	+	(+)	+	+	+	+	+	+	+	+	(+)
Criterion 3											
Slope OLS	−	+	−	−	+	+	+	+	−	+	−
Slope PGLS	−	+	−	−	+	+	+	+	−	+	−
Criterion 4 (OLS, *R* ^2^)	−	+	+	−	+	+	+	+	−	+	−
*Anura*											
Criterion 1 (variance ratio)	+	−	+	−	+	−	+	+	+	+	+
Criterion 2											
Unimodality	−	+	−	−	+	+	+	+	−	+	−
95% within ±2 *SD*	−	+	+	+	+	+	−	−	+	+	(+)
Criterion 3											
Slope OLS	−	+	−	−	+	+	+	+	−	+	−
Slope PGLS	−	+	−	−	+	+	+	+	−	+	−
Criterion 4 (OLS, R^2^)	−	−	+	−	+	+	+	+	−	+	−
*Caudata*											
Criterion 1 (variance ratio)	+	−	+	+	+	−	+	+	+	+	+
Criterion 2											
Unimodality	−	+	−	+	+	+	+	+	+	+	+
95% within ±2 *SD*	(+)	+	+	+	+	+	−	(+)	+	+	−
Criterion 3											
Slope OLS	−	NA	−	+	+	NA	+	+	−	+	+
Slope PGLS	+	NA	−	+	+	NA	+	+	−	+	−
Criterion 4 (OLS, *R* ^2^)	−	NA	−	+	+	NA	+	−	−	+	+
*Reptiles*											
Criterion 1 (variance ratio)	−	−		−	−	−	−		−		−
Criterion 2											
Unimodality	+	+		+	−	+	+		+		+
95% within ±2 *SD*	+	+		+	+	+	+		+		+
Criterion 3											
Slope OLS	−	−		−	+	−	−		−		−
Slope PGLS	−	−		−	+	−	−		−		−
Criterion 4 (OLS, *R* ^2^)	−	−		−	+	−	−		−		−

Note

The database on reptilian species traits is from Hallmann and Griebeler (2018). Criteria on invariance of traits are based on Price et al. (2014). Criteria (1) and (2) refer to type I invariance and criteria (3) through (4) to type II invariance (Savage et al., 2006).

Abbreviations: ASM, age at (sexual) maturity; BW, birthweight; CPA, clutches per year (only reptiles); CS, clutch size; EM, egg mass; I, incubation time; L, larval period (only amphibians); ML, maximum longevity; MS, metamorphosis size (only amphibians); NA, not available; OS, offspring size (only amphibians); RO, reproductive output (only amphibians); SSM, size at (sexual) maturity.

Criterion 1 = low variance in the life history trait compared to that seen in body mass; Criterion 2 = unimodal distribution or 95% of the observations are within plus/minus two standard deviations (**±**2 *SD*) of the mean; Criterion 3 = small regression slope under ordinary linear least squares analysis (OLS) or phylogeny‐informed regression analysis (PGLS); Criterion 4 = low coefficient of determination when regressing the trait against body mass (OLS, *R* ^2^). For details on criteria (1) and (2), refer to Table 2, and for details on criteria (3) and (4) to Table 3. + = criterion fulfilled for this trait, (+) = criterion marginally fulfilled (i.e., >94% of values within ± 2 *SD* percentage), ‐ = criterion not fulfilled.

### 3.1 Invariance of life history traits in amphibians

Criterion (1)—low variance in the species trait compared to that seen in body mass (type I, Figure 1, Table 2). When analyzing all amphibians together, body mass variance was larger than that seen in the life history trait for eight (age at maturity, offspring size, reproductive output, incubation time, larval period, metamorphosis size, maximum longevity, size at maturity) out of the eleven traits studied (Table 2). When analyzing Anura and Caudata separately, both orders resembled the results obtained for all amphibians. The only exception was that in Caudata clutch size was invariant, but neither in Anura nor in all amphibians (Table 2).

**Table 2:** Detailed results on criteria (1) and (2) on type I invariance of life history traits

	Age at maturity	Birthweight	Offspring size	Clutch size	Reproductive output	Egg mass	Incubation time	Larval period	Max. longevity	Metamorphosis size	Size at maturity
*All amphibians*											
Variance	0.07	1.68	0.06	0.75	<0.01	1.17	0.41	0.12	0.12	0.06	0.13
Body mass var /trait var	**8.48**	0.34	**9.67**	0.77	**142.98**	0.49	**1.40**	**4.84**	**4.79**	**10.12**	**4.28**
Hartigan's dip test D (*p*‐value)	0.06 (<0.01)	0.08 (**0.43**)	0.04 (<0.01)	0.02 (<0.01)	<0.01 (**0.99**)	0.07 (**0.64**)	0.04 (**0.81**)	0.05 (**0.45**)	0.04 (<0.01)	0.06 (**0.12**)	0.04 (<0.01)
% within ± 2 *SD*	**95.49**	94.74	**95.86**	**97.67**	**98.65**	**100**	**96.47**	**95.45**	**96.47**	**98.51**	94.88
*Anura*											
Variance	0.07	1.58	0.05	0.76	<0.01	0.75	0.17	0.02	0.13	0.06	0.14
Body mass var/trait var	**7.89**	0.36	**10.24**	0.74	**134.21**	0.75	**3.27**	**27.80**	**4.50**	**8.66**	**4.05**
Hartigan's dip test D (*p*‐value)	0.07 (<0.01)	0.08 (**0.79**)	0.04 (<0.01)	0.02 (<0.01)	<0.01 (**0.99**)	0.10 (**0.36**)	0.05 (**0.62**)	0.06 (**0.57**)	0.05 (<0.01)	0.08 (**0.09**)	0.05 (<0.01)
% within ± 2 *SD*	93.07	**100**	**95.54**	**97.34**	**98.57**	**100**	93.48	92.31	**95.20**	**97.22**	94.81
*Caudata*											
Variance	0.04	1.75	0.04	0.41	<0.01	2.07	0.37	0.22	0.07	0.05	0.04
Body mass var/trait var	**18.16**	0.36	**16.56**	**1.56**	**228.04**	0.31	**1.71**	**2.84**	**9.17**	**3.36**	**15.02**
Hartigan's dip test D (*P*‐value)	0.06 (<0.01)	0.13 (**0.25**)	0.05 (<0.01)	0.02 (**0.70**)	<0.01 (**0.99**)	0.13 (**0.35**)	0.05 (**0.60**)	0.08 (**0.47**)	0.04 (**0.19**)	0.05 (**0.68**)	0.04 (**0.19**)
% within ± 2 *SD*	94.64	**100**	**97.13**	**95.50**	**99.24**	**100**	92.31	94.44	**95.71**	**96.77**	93.57
*Reptiles—HG2018*											
Variance	0.13	0.61	–	0.25	0.09	0.77	0.04	–	0.08	–	0.29
Body mass var/trait var	0.06	0.29	–	0.12	0.05	0.30	0.02	–	0.06	–	0.15
Hartigan's dip test D (*p*‐value)	0.04 (**0.08**)	0.04 (**0.60**)	–	0.03 (**0.13**)	0.06 (0.01)	0.04 (**0.73**)	0.02 (**0.85)**	–	0.02 (**0.52**)	–	0.04 (**0.82**)
% within ± 2 *SD*	**99.17**	**98.72**	**–**	**95.28**	**95.62**	**96.77**	**97.16**	**–**	**99.29**	**–**	**100**

Note

Criteria were applied to all amphibians, the amphibian orders Anura and Caudata, and the reptiles. For reptiles, the dataset published by Hallmann and Griebeler (2018) was analyzed (HG2018). Variance = variance of the life history trait; body mass var/trait var = ratio of the body mass variance and life history trait variance (both standardized, see main text); Hartigan's dip test D (*p*‐value) = D and *p*‐value of this test on unimodality of the trait distribution; % within ± 2 *SD* = percentage of trait values within plus/minus two standard deviations (*SD*) of the mean. All traits were log_10_‐transformed prior to the application of a criterion. Bold highlights values consistent with type I invariance. Please note that our amphibian dataset assesses annual clutch frequency as reproductive output (maximum number of reproduction events per year), whereas the HG2018 dataset as number of clutches per year. The amphibian dataset further assesses size as birthweight and offspring size, whereas the HG2018 dataset provides only birthweight.

Criterion (2)—unimodal distribution of life history traits with 95% of values observed within ± 2 *SD* of the mean (type I, Table 2). For all amphibians, unimodality was observed for six traits (birthweight, reproductive output, egg mass, incubation time, larval period, and metamorphosis size). Nine of the eleven traits studied passed and the remaining two others birthweight and size at maturity marginally failed the 95% of values observed within ± 2 *SD* of the mean criterion (for histograms, see Figure 1; for Hartigan's dip test, see Table 2).

For the Anura, tests on the unimodality of trait values indicated again invariance for the six traits that passed this criterion for all amphibians. Out of these, the 95% of values observed within ± 2 *SD* of the mean criterion corroborated invariance only for birthweight, reproductive output, egg mass, and metamorphosis size, but not for incubation time and larval period. The application of the 95% of values observed within ± 2 *SD* of the mean criterion indicated invariance for offspring size, clutch size, maximum longevity, and potentially for size at maturity all having not passed the unimodality criterion (Tables 1 and 2).

When analyzing the Caudata, for nine traits a unimodal distribution was not rejected (Tables 1 and 2). These were the six traits already showing such a distribution in all amphibians and in Anura, and the three traits clutch size, maximum longevity, and size at maturity. Except for incubation time, and size at maturity, all these nine traits also passed or at least marginally passed (larval period) the criterion that 95% of values are within ± 2 *SD* of the trait mean (Tables 1 and 2) in Caudata.

Criterion (3)—low coefficient of determination (*R^2^*) and criterion (4)—small slope of OLS and PGLS linear models (type II, Table 3). When analyzing all amphibians together, six traits met each of our three criteria on type II invariance (*R* ^2^, OLS slope, and PGLS slope; Tables 1 and 3). These were birthweight, reproductive output, egg mass, incubation time, larval period, and metamorphosis size. *R* ^2^ further indicated invariance of offspring size in all amphibians.

**Table 3:** Detailed results on criteria (3) and (4) on type II invariance of life history traits

	Trait	DF	*R* ^2^	Adj. *R* ^2^	p	Slope	CI_s_	Intercept	CI_i_	*λ*
*Amphibians*										
OLS	Age at maturity	209	.07	.06	<.01	0.08	0.04, 0.12	0.28	0.22, 0.33	–
PGLS		188	–	–	<.01	0.09	0.05, 0.09	0.20	−0.11, 0.20	0.90
OLS	Birthweight	16	**<.01**	−.06	**.83**	0.13	−1.07, 1.33	0.20	−1.31, 1.71	–
PGLS		16	–	–	**.81**	−0.17	−1.69, 1.34	0.59	−1.74, 2.91	0.58
OLS	Offspring size	402	**.02**	**.02**	<.01	0.05	0.02, 0.08	0.22	0.19, 0.26	
PGLS		327			<.01	0.05	0.02, 0.09	0.40	0.09, 0.72	0.92
OLS	Clutch size	445	.18	.18	<.01	0.48	0.39, 0.57	1.97	1.85, 2.09	–
PGLS		365	–	–	<0.01	0.30	0.21, 0.40	1.29	0.34, 2.25	0.91
OLS	Reproductive output	553	**<.01**	<.01	**.95**	−0.01	−0.01, 0.01	0.02	0.01, 0.03	
PGLS		445	–	–	**.92**	<0.01	−0.01, 0.01	0.92	0.77, 1.07	0.81
OLS	Egg mass	14	**<.01**	−.07	**.94**	−0.03	−0.89, 0.82	−1.62	−2.80, −0.45	–
PGLS		14	–	–	**.74**	−0.23	−1.68, 1.21	−1.39	−1.35, −1.30	1.00
OLS	Incubation time	60	**<.01**	<.01	**.97**	<0.01	−0.20, 0.21	1.30	1.01, 1.59	–
PGLS		57	–	–	**.06**	0.19	−0.01, 0.38	0.97	0.16, 1.77	0.95
OLS	Larval period	39	**<.01**	−.02	**.80**	0.02	−0.19, 0.24	1.96	1.71, 2.20	–
PGLS		39	–	–	**.53**	0.08	−0.17, 0.33	2.06	1.52, 2.60	0.76
OLS	Max. longevity	209	.13	.12	<.01	0.15	0.10, 0.20	0.77	0.69, 0.85	–
PGLS		189	–	–	<.01	0.19	0.14, 0.19	0.38	0.69, 0.99	0.70
OLS	Metamorphosis size	46	**.04**	.02	**.18**	−0.08	−0.19, 0.03	1.49	1.35, 1.63	–
PGLS		41	–	–	**.72**	−0.03	−0.20, 0.14	1.39	1.05, 1.74	0.63
OLS	Size at maturity	211	.08	.08	<.01	0.13	0.07, 0.19	0.83	0.74, 0.92	–
PGLS		191	–	–	<.01	0.18	0.11, 0.17	0.45	0.45, 0.76	0.59
*Anura*										
OLS	Age at maturity	139	.14	.13	<.01	0.12	0.07, 0.16	0.16	0.09, 0.23	–
PGLS		124	–	–	<.01	0.11	0.05, 0.17	0.16	−0.14, 0.46	0.91
OLS	Birthweight	10	.19	.10	**.18**	−1.01	−2.95, 0.34	1.99	0.05, 0.34	–
PGLS		10	–	–	**.05**	−1.31	−2.65, 0.02	2.23	0.03, 4.44	0.91
OLS	Offspring size	344	**.02**	.01	.02	0.04	0.01, 0.07	0.20	0.16, 0.24	
PGLS		274	–	–	<.01	0.05	0.02, 0.09	0.35	0.10, 0.60	0.89
OLS	Clutch size	378	.28	.27	<.01	0.58	0.46, 0.67	2.00	1.88, 2.11	–
PGLS		304	–	–	<.01	0.35	0.24, 0.46	1.78	1.00, 2.56	0.88
OLS	Reproductive output	476	**<.01**	<.01	**.74**	<0.01	−0.01, 0.02	0.02	0.01, 0.04	
PGLS		373	–	–	**.86**	<0.01	0.00, 0.01	0.03	−0.25, 0.30	1.00
OLS	Egg mass	9	**<.01**	−.12	**.92**	−0.05	−1.07, 0.96	−1.53	−3.20, 0.14	–
PGLS		9	–	–	**.73**	0.11	−0.63, 0.84	−1.49	−2.61, −0.36	0.88
OLS	Incubation time	31	**.01**	−.02	**.58**	−0.07	−0.31, 0.17	0.98	0.67, 1.30	–
PGLS		31	–	–	**.13**	0.25	−0.08, 0.57	0.51	−0.12, 1.14	0.87
OLS	Larval period	22	**.01**	−.03	**.61**	−0.04	−0.17, 0.10	1.90	1.73, 2.07	–
PGLS		22	–	–	**.72**	−0.03	−0.18, 0.12	1.89	1.62, 2.17	0.94
OLS	Max. longevity	142	.23	.23	<.01	0.21	0.14, 0.24	0.60	0.50, 0.69	–
PGLS		125	–	–	<0.01	0.22	0.15, 0.22	0.75	0.46, 1.05	0.79
OLS	Metamorphosis size	30	**<.01**	−.04	**.76**	−0.03	−0.22, 0.16	1.39	1.11, 1.69	–
PGLS		22	–	–	**.55**	−0.07	−0.32, 0.17	1.41	1.06, 1.77	0.14
OLS	Size at maturity	144	.17	.16	<.01	0.20	0.13, 0.27	0.63	0.53, 0.75	–
PGLS		127	–	–	<.01	0.21	0.13, 0.29	0.82	0.53, 1,11	0.59
*Caudata*										
OLS	Age at maturity	68	.06	.05	.03	0.05	0.01, 0.10	0.44	0.37, 0.51	–
PGLS		64	–	–	**.13**	0.04	−0.01, 0.10	0.42	0.16, 0.68	0.78
OLS	Birthweight	6	–	–	–	–	–	–	–	–
PGLS		6	–	–	–	–	–	–	–	–
OLS	Offspring size	56	.08	.06	.04	0.07	0.01, 0.14	0.40	0.30, 0.48	–
PGLS		53	–	–	.04	0.07	0.01, 0.13	0.44	0.01, 0.87	1.00
OLS	Clutch size	65	**.04**	.03	**.08**	0.18	−0.02, 0.38	1.57	1.28, 1.00	–
PGLS		61	–	–	**.30**	0.11	−0.11, 0.33	1.51	−0.04, 3.05	1.00
OLS	Reproductive output	75	**<.01**	−.01	**.79**	−0.01	−0.04, 0.03	−0.01	−0.06, 0.04	–
PGLS		72	–	–	**.05**	0.07	0.00, 0.13	0.44	0.01, 0.88	1.00
OLS	Egg mass	5	–	–	–	–	–	–	–	–
PGLS		5	–	–	–	–	–	–	–	–
OLS	Incubation time	27	**.01**	−.03	**.58**	0.06	−0.15, 0.27	1.69	1.38, 2.01	–
PGLS		26	–	–	**.35**	0.10	−0.12, 0.32	1.53	0.85, 2.21	1.00
OLS	Larval period	18	.17	.11	**.09**	0.38	−0.04, 0.80	1.85	1.47, 2.23	–
PGLS		17	–	–	**.37**	0.23	−0.30, 0.75	2.26	1.49, 3.04	0.57
OLS	Max. longevity	65	.10	.09	<.01	0.09	−0.01, 0.16	1.07	0.97, 1.16	–
PGLS		64	–	–	<.01	0.13	0.05, 0.21	0.86	0.60, 1.12	0.44
OLS	Metamorphosis size	22	**<.01**	−.04	**.84**	−0.02	−0.24, 0.19	1.49	1.31, 1.66	–
PGLS		19	–	–	**.88**	0.02	−0.20, 0.24	1.37	1.08, 1.65	0.95
OLS	Size at maturity	65	**.02**	<.01	**.22**	0.05	−0.03, 0.13	1.16	1.04, 1.28	–
PGLS		64	–	–	.04	0.10	0.01, 0.19	0.96	0.68, 1.25	0.33
*Reptiles—HG2018*										
OLS	Age at maturity	118	.65	.64	<.01	0.20	0.17, 0.22	2.60	2.52, 2.68	–
PGLS		119	–	–	<.01	0.19	0.08, 0.16	2.82	2.66, 2.98	0.55
OLS	Birthweight	75	.69	.68	<.01	0.44	0.37, 0.51	−0.40	−0.60, −0.19	–
PGLS		77	–	–	<.01	0.41	0.32, 0.51	−0.36	−0.68, −0.04	0.26
OLS	Clutch size	209	.47	.47	<.01	0.24	0.20, 0.27	0.31	0.21, 0.41	–
PGLS		208	–	–	<.01	0.23	0.19, 0.28	0.37	0.11, 0.63	0.77
OLS	Clutches p.a.	134	**<.01**	−.01	**.86**	0.01	−0.03, 0.04	0.23	0.14, 0.31	–
PGLS		134	–	–	**.97**	−0.01	−0.05, 0.05	0.29	0.07, 0.51	0.67
OLS	Egg mass	59	.90	.90	<.01	0.52	0.47, 0.57	−0.52	−0.68, −0.37	–
PGLS		61	–	–	<.01	0.45	0.37, 0.53	−0.34	−0.65, 0.04	0.85
OLS	Incubation time	173	.10	.09	<.01	0.04	0.02, 0.06	1.79	1.74, 1.85	–
PGLS		174	–	–	**.06**	0.03	−0.01, 0.06	1.82	1.67, 1.96	0.72
OLS	Max. longevity	277	.39	.38	<.01	0.15	0.13, 0.17	0.85	0.79, 0.91	–
PGLS		274	–	–	<.01	0.13	0.10, 0.16	0.92	0.75, 1.09	0.73
OLS	Size at maturity	51	.60	.59	<.01	0.29	0.23, 0.36	0.70	0.47, 0.94	–
PGLS		53	–	–	<.01	0.27	0.20, 0.34	0.75	0.46, 1.04	0.93

Note

Criteria were applied to all amphibians, the amphibian orders Anura and Caudata, and the reptiles. For reptiles, the results from Hallmann and Griebeler (2018) are shown (HG2018). Criteria were explored under ordinary least squares regression analysis (OLS, Price et al., 2014) and phylogeny‐informed regression analysis (PGLS) on log_10_‐log_10_‐transformed data. DF = degrees of freedom; *R* ^2^ = coefficient of determination (<.05 indicates invariance, Price et al., 2014); Adj *R* ^2^ = adjusted *R* ^2^; *p* = *p*‐value (≥.05 indicates invariance); CI_s_ = 95% confidence interval of slopes; CI_i_ = 95% confidence interval of intercepts. Lambda (λ) values rate the impact of the phylogeny on studied relationships between life history traits and body mass (residuals). Please note that adjusted *R* ^2^ can drop below zero. This happens if *R* ^2^ is zero or close to zero and usually indicates that the model fits poorly the data. Bold highlights values consistent with type II invariance. – = not applicable under PGLS or sample size too small.

For the Anura, five out of the six traits passing all our three criteria on invariance (*R* ^2^, OLS slopes, and PGLS slopes) in all amphibians passed them again. These were reproductive output, egg mass, incubation time, larval period, and metamorphosis size. Both OLS and PGLS slopes indicated invariance for birthweight fulfilling also the *R^2^* criterion in all amphibians. *R* ^2^ indicated invariance for offspring size, but in Anura neither the OLS nor the PGLS slope indicated an invariance of this trait (Tables 1 and 3).

For the Caudata, neither OLS nor PGLS regressions could be established on birthweight and egg mass due to small sample sizes (Table 3). The four traits clutch size, reproductive output, incubation time, and metamorphosis size met all our three criteria on invariance (*R* ^2^, OLS slopes, and PGLS slopes). Both the OLS and PGLS slope further indicated invariance for larval period, the OLS slope for age at maturity, and both the OLS slope and *R^2^* for size at maturity.

Thus, in all amphibians, Anura and Caudata reproductive output, incubation time, and metamorphosis size were invariant based on each of our four criteria. Clutch size was invariant in Caudata, but not in Anura and in all amphibians. Birthweight was only invariant in all amphibians (Tables 1 and 3).

Criterion (5)—isometric relation (i.e., the slope is unity in a log–log plot) between two life history traits (type II, Tables 4 and S2). Linear standardized major axis (SMA) regression models established for pairs of life history traits (criterion 5) demonstrated an isometric relation between age at maturity and offspring size for all amphibians and the Caudata. For the Caudata, SMA further indicated an isometric relation between age at maturity and metamorphosis size (Table 4).

**Table 4:** Detailed results on criterion (5) on type II invariance of life history traits. Criterion (5) was applied to all amphibians, the amphibian orders Anura and Caudata, and the reptiles

	*R* ^2^	*p*	Slope	CI_s_	Intercept	CI_i_
*Amphibians*						
Age at maturity ~ offspring size	.12	<.01	1.03	0.93, 1.15	−0.01	−0.06, 0.04
*Caudata*						
Age at maturity ~ offspring size	.03	.05	0.92	0.77, 1.09	<0.01	−0.08, 0.10
Reproductive output ~ metamorphosis size	.37	.05	1.22	0.70, 2.17	−1.04	−2.70, 0.61
*Reptiles—HG2018*						
Birthweight ~ egg mass	.60	<.01	0.94	0.75, 1.19	0.13	−0.13, 0.38
Age at maturity ~ maximum longevity	.48	<.01	1.06	0.91, 1.23	1.74	1.52, 1.96

Note

For reptiles, the dataset published by Hallmann and Griebeler (2018) was analyzed (HG2018). Only those trait combinations are shown for which the SMA slope differs significantly from zero, and the 95% confidence interval of the slope includes unity. For SMA results on all trait combinations, refer to Table S2. Results of SMA regressions analysis: *R* ^2^ = coefficient of determination; *p* = *p*‐value; CI_s_ = 95% confidence interval of slopes; CI_i_ = 95% confidence interval of intercepts.

### 3.2 Invariance of life history traits in reptiles

Criterion (1)—low variance in the species trait compared to that seen in body mass (type I, Table 2). None of the life history traits had a smaller variance than that of body mass refuting any invariance for all traits under study based on this criterion (Tables 1 and 2).

**Figure 2:**
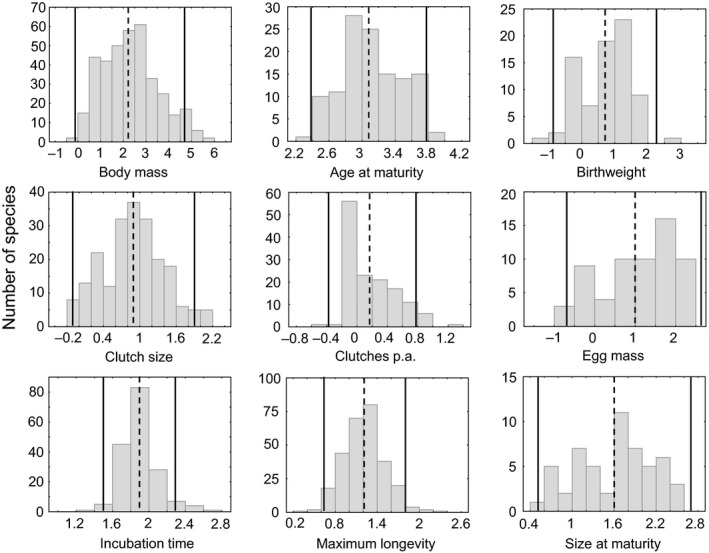
Histograms of log_10_‐transformed data for each of the reptilian life history traits studied (criteria 1 and 2, type I invariance, data from Hallmann & Griebeler, 2018). In all plots, the dashed line represents the mean value of each distribution and the two solid lines represent plus/minus two standard deviations (±2 *SD*) of the mean. N_body mass_ = 369, N_age at maturity_ = 121, N_birthweight_ = 78, N_clutch size_ = 210, N_clutches p.a._ = 137, N_egg mass_ = 62, N_incubation time_ = 174, N_maximum longevity_ = 280, N_size at maturity_ = 54

Criterion (2)—unimodal distribution of life history traits with 95% of values observed within ± 2 *SD* of the mean (type I, Figure 2, Table 2). All traits studied had unimodal distributions (for Hartigan's dip test results, see Table 2), except for clutches p.a. For all eight life history traits studied including clutches p.a., at least 95% of observations fell within ± 2 *SD* of the mean (Tables 1 and 2).

Criterion (3)—low coefficient of determination (*R^2^*) and criterion (4)—small slope of OLS and PGLS linear models (type II, Table 3). For the traits age at maturity, birthweight, clutch size, egg mass, maximum longevity, and size at maturity, the coefficients of determination (*R^2^*) of OLS models, OLS slopes, and PGLS slopes indicated no invariance. Contrary, all these three criteria supported invariance of clutches p.a. The nonsignificant PGLS slope further indicated invariance of incubation time (Tables 1 and 3).

Criterion (5)—isometric relation (i.e., the slope is unity in a log–log plot) between two life history traits (type II, Table 4). Under SMA, an isometric relationship was observed between birthweight and egg mass and between age at maturity and maximum longevity (Tables 4 and S2).

## 4 DISCUSSION

### 4.1 Invariances in amphibian and reptilian life history traits

Biological traits are inherently variable. This is why Price et al. (2014) use the terms “approaching invariant” or “effectively invariant” for invariance and elaborated with their five criteria a framework for deciding whether a trait is invariant under body size transformation or not. However, we regard herein a trait passing each of all criteria on the respective invariance type (type I, type II, or type I + II) as being invariant under body size transformation, as this is the best evidence that we can have under this framework (see 4.2). Type I invariance was indicated by criteria (1) and (2) for reproductive output, incubation time, larval period, and metamorphosis size in all amphibians, for reproductive output and metamorphosis size in Anura, and for clutch size, reproductive output, larval period (only 94.44% of observations are within ± 2 *SD* of the mean), metamorphosis size, and maximum longevity in Caudata (Tables 1 and 2). Type II invariance was suggested by criteria (3) and (4) for egg mass, reproductive output, birthweight, incubation time, larval period, and metamorphosis size in all amphibians, for egg mass, reproductive output, incubation time, larval period, and metamorphosis size in Anura, and for clutch size, reproductive output, incubation time, and metamorphosis size in Caudata (Tables 1 and 3). Type I + II invariance was thus suggested for reproductive output, incubation time, larval period, and metamorphosis size in all amphibians, for reproductive output and metamorphosis size in Anura, and for clutch size, reproductive output, and metamorphosis size in Caudata (Tables 1, 2, 3). Whether a trait was classified as invariant with respect to the three different types of invariance thus differed across amphibian groups studied. In reptiles, however, no trait passed criteria (1) and (2) on type I invariance, only clutches p.a. passed criteria (3) and (4) on type II invariance, and thus, no trait was type I + II invariant (Tables 1, 2, 3).

Amphibians. The traits being invariant according to any of the three types of invariance (types I, II, and I + II) in the amphibian groups studied cover different phases within an amphibian's life. The considerable variation among (and even within) species in the duration of the egg and larval/tadpole phase (Figure 1, Werner, 1986) facilitating development in aquatic or terrestrial habitats may explain that the life history traits incubation time, larval period, and metamorphosis size are independent of adult body mass. The type I + II invariance of larval duration and metamorphosis size of a larva/tadpole in all amphibians, Anura, and Caudata could, in addition, reflect the strong changes during metamorphosis leading to morphologically and ecologically very divergent adults (Dodd & Dodd, 1976; Fritzsch, 1990; Laudet, 2011; Lynn, 1961). That reproductive output is type I + II invariant in all amphibians, in Anura and in Caudata, conforms to observations on other vertebrates. This study shows that clutches p.a. is type I + II invariant in reptiles (Tables 1, 2, 3). Birds (mean = 1.02, *SD* = 0.15) and mammals (mean = 1.15, *SD* = 0.81) show a small variability in their number of clutches/litters per year (data from Werner & Griebeler, 2011), and to the best of our knowledge, no allometric regression on this trait has been published so far for these vertebrates. By contrast, that egg mass is type II invariant in all amphibians and in Anura (the sample size on Caudata was too small) is surprising. Dol'nik (2000) had established an allometric regression on egg mass for amphibians, and egg mass is generally associated with body mass in other vertebrates (e.g., Hallmann & Griebeler, 2018; Hendriks & Mulder, 2008; Peters, 1983). As our sample size on amphibian egg mass is very small (*n* = 19), we cannot exclude that this and/or potential differences in scaling of egg mass to body mass seen between Anura and Caudata or even between families and genera could have inflated the confidence interval of the estimated slope and that this hampered a finding of a significant scaling exponent (Table 3). Evidence for scaling of egg mass to body mass comes from offspring size which is expected to correlate to egg mass (Nee et al., 2005) and for which our amphibian sample size is large. Offspring size is neither type I nor type II invariant in all amphibians, Anura, and Caudata (Tables 1, 2, 3). The latter argument further questions type II invariance of birthweight in all amphibians (which is again based on a small sample size) as birthweight should relate to offspring size, too. However, the large variability in amphibian reproductive modes could contradict this line of argument against the invariance of egg mass and birthweight. Similar‐sized species laying eggs into the water should have smaller eggs than species having terrestrial eggs, and the aquatic clutches should be larger than terrestrial ones. Likewise, for similar‐sized species birthweight should be smaller for oviparous species than for similar‐sized ovoviviparous or viviparous species. A test of the hypothesis on invariance of egg mass and birthweight based on these expectations requires considerably larger sample sizes than ours and a good coverage of different reproductive modes seen in amphibians.

The orders Anura and Caudata differed in traits classified as invariant according to the different criteria, except for reproductive output and metamorphosis size being type I + II invariant in both orders (see above). Clutch size and maximum longevity were type I invariant in Caudata, but not in Anura. Clutch size was also type II invariant in Caudata, but not in Anura, and larval period was type II invariant in Anura, but not in Caudata (Tables 1, 2, 3). The type II invariance of larval period in Anura, but not in Caudata, could indeed reflect differences in metamorphosis seen between anuran and caudatan species. In anurans, the ancestral biphasic life with a aquatic phase before metamorphosis and a terrestrial phase after metamorphosis is more frequent than in salamanders and the metamorphosis is associated with stronger morphological and physiological changes in the Anura than in Caudata (Lynn, 1961). Reproductive modes influencing egg and larval period are more diverse in frogs than in salamanders (Lynn, 1961). Consistent with this, our results show that the body mass to larval period variance (type I invariance) is an order of magnitude larger in Anura (27.80) than in Caudata (2.84). This supports a type II invariance of larval period in Anura, but not in Caudata.

We believe that the remaining differences seen in invariances of traits between Anura and Caudata are most probable statistical artifacts. Type I invariance of clutch size and maximum longevity in Caudata, but not in Anura, is not supported by a considerable difference in trait to body mass variances seen between the two amphibian orders. The clutch size to body mass variance is close to unity in both orders, and in Caudata (1.56), it is only about twice as high as that seen in Anura (0.74). Although being larger than unity, the ratio of maximum longevity and body mass is small compared to that of other invariant traits (Table 2) and also only about twice as high in Caudata (9.17) than in Anura (4.50). Together with the fact that our test on the unimodality of trait distributions is conservative (type 1 error is minimized, and the null hypothesis of this test assumes no deviation from unimodality), both question that clutch size and maximum longevity are type I invariant only in Caudata, but not in Anura and in all amphibians. A considerable impact of body mass on maximum longevity in Caudata is further indicated by the absence of a type II invariance in this order.

That clutch size is type II invariant in Caudata but not in Anura is questioned by several observations. OLS and PGLS slope estimates are all considerably larger than zero in Anura and in Caudata and at least the OLS slope estimated for Caudata was marginally significant (Table 3). Our dataset on clutch size against body mass was a magnitude smaller in Caudata than in Anura. Finally, allometric relationships between clutch size and body mass had already been established in amphibians by other authors (Dol'nik, 2000; Duellman & Trueb, 1994).

With respect to criterion (5) (Table 4), our dataset indicated isometry for age at maturity and offspring size for all amphibians and Caudata. An isometry between reproductive output and metamorphosis size was only observed for Caudata (Table 4). We believe that all these invariances are statistical artifacts for the following reasons. For all amphibians, the 95% confidence intervals of OLS and PGLS regression slopes on age at maturity (offspring size) and body mass do overlap (Table 3). They indicate that age at maturity and offspring size scale with similar exponents to body mass. SMA regressions suggest different slopes in both amphibian orders (Anura: 1.23, Caudata: 0.92), and thus, an intermediary slope close to unity (1.03) could result when pooling them to all amphibians (Table 4). Consistent with this, 95% confidence intervals of SMA slopes of Anura and Caudata do barely overlap (Anura: 1.06, 1.44; Caudata: 0.77, 1.09). The isometry of reproductive output and metamorphosis size in Caudata is based on a very broad 95% confidence interval of the SMA slope (0.70, 2.17), whereas the slope value itself is 1.22 and thus considerably larger than unity (Table 4).

Reptiles. In reptiles, no trait was type I invariant and thus no trait was type I + II invariant. Out of all eight reptilian traits studied, only clutches p.a. turned out to be type II invariant. All this indicates a large impact of body size on life history traits in reptiles as other studies have already observed before (see Dol'nik, 2000; Dunham & Miles, 1985; Fitch, 1970; Hallmann & Griebeler, 2015, 2018; Peters, 1983; Scharf et al., 2014; Schmidt‐Nielsen, 1984). Within reptiles, type I invariance of clutch size was shown for anoles and eublepharid gekkotan species (Kratochvíl & Kubička, 2007). These lizards lay only one or two eggs, which is exceptional in reptiles as a variable clutch size is ancestral to this taxon (Kratochvíl & Kubička, 2007), and indicates that anoles and eublepharid gekkotan species almost fill up their entire capacity of the body cavity with eggs (Meiri, Feldman, & Kratochvil, 2014). The invariance of clutch size in anoles and eublepharid gekkotan species (Kratochvíl & Kubička, 2007) shows that we cannot exclude that some life history traits could be invariant at lower taxonomic levels within the reptile class. Likewise, differences seen in invariances of traits between Anura and Caudata also point to an impact of the taxonomic level studied on whether a trait is invariant or not. However, as species sample sizes decrease when studying such reptilian subgroups, invariances are difficult to demonstrate (see below). The latter generally applies to any subgroup (e.g., defined by taxonomy, reproductive mode, or habitat use) with a small species sample.

In reptiles, criterion (5) indicated isometry for egg mass and birthweight as well as for age at maturity and maximum longevity. These isometries are also most probably artifacts. The 95% confidence intervals of OLS and PGLS slopes (Table 3) overlap, which indicates no significant difference in scaling exponents for both traits with body mass. Moreover, both isometries conform to the situation that one trait is a fraction of the other which is not a true invariance (Nee et al., 2005). That in oviparous and ovoviviparous species, birthweight is a fraction of egg mass is obvious, as the hatchling had consumed energy from the egg. For age at maturity, Shine and Charnov (1992) have shown in lizards and snakes that it is inversely proportional to adult mortality rate, which in turn is inversely proportional to maximum longevity.

Comparison of amphibians and reptiles. Overall, our results on invariances of life history traits suggest that (adult) size matters much in reptiles and that it most probably considerably shaped the evolution of their life histories, whereas going through a metamorphosis (what the majority of amphibian species do) and the evolutionary trend toward independence of egg and larval/tadpole development from the water additionally constrained life history evolution in amphibians. The latter manifests in that out of all traits studied, only clutches p.a. is invariant (type II) in reptiles. In amphibians, reproductive output (a proxy of clutches p.a.), incubation time, larval period, and metamorphosis size are type I or type II invariant either in amphibians or in the orders Anura and Caudata. That in reptiles, body mass is a main driver of life history evolution could be a consequence of their fully terrestrial lifestyle (Collar et al., 2011) being enabled by the advent of the amniotic egg (Sander, 2012; Sumida & Martin, 1997; Morrow et al., 2019). The amniotic egg contains membranes improving gas exchange. It allows the production of larger eggs compared to similar‐sized nonamniotes and of more developed hatchlings (Thompson & Russell, 1998). Thus, adult life span divided by the time needed to reach maturity (time before reproduction vs. time available for reproduction) is on average smaller in amphibians than in reptiles (Morrow et al., 2019). It increases significantly with body mass in Squamata but is independent of body mass in amphibians (Morrow et al., 2019). These observations are consistent with that incubation time, larval period, and metamorphosis size (each of these traits contributes to the time that an organism spends to prepare to reproduce) turned out to be invariant toward body size in amphibians, but not in reptiles. Likewise, Morrow et al. (2019) observed that lifetime reproductive effort and relative offspring size decrease with body mass in amphibians and squamates (although in squamates, this decrease is not significant for lifetime reproductive effort). Their two observations also strengthen our results. Lifetime reproductive effort is related among others to egg mass, clutch size, reproductive output, and birthweight. While reproductive output (clutches p.a.) turned out to be invariant in amphibians and reptiles (and most probably also in birds and mammals, see above) under all criteria, clutch size scales to body mass in amphibians and reptiles. Egg mass and birthweight also scale to body mass in reptiles and most probably also in amphibians (as discussed above, Tables 1 and 3). With respect to relative offspring size defined as the ratio of offspring size and adult body mass, we found that the trait offspring size/birthweight is not type II invariant in amphibians and reptiles (Table 1). This observation is consistent with that Morrow et al. (2019) found a decrease in relative offspring size in amphibians and reptiles. The larger number of life history traits being invariant toward body size in amphibians than in reptiles could further reflect potential niche differences between eggs, larvae/tadpoles, and adults (except for the species with young sharing the habitat of adults). An organism having more life history stages tolerates a larger environmental variability (Wingfield, 2008). This would be consistent with that amphibian species having a more complex life cycle than reptiles possess an evolutionary adaptation to the different environmental conditions experienced during their life. In amphibians, larval growth and development show a high plasticity toward food availability and their timing of metamorphosis reflects a trade‐off between growth and predation risk (Werner, 1986).

### 4.2 Power of the five criteria to identify invariant life history traits

The five criteria presented in Price et al. (2014) address and solve some problems discussed in the context of the theory on life history invariants (Charnov, 1993; Nee et al., 2005; Savage et al., 2006). However, our study overcame some of these but also identified new ones.

Identification of type I invariant life history traits. Criteria (1) and (2) address type I invariance. Price et al. (2014) proposed that invariant life history traits should have a lower variance compared to that seen in body mass (criterion 1). These authors already mentioned a shortcoming of this criterion, which we successfully tackled by standardizing variances. Our procedure is based on their notion of a “guideline for exactly how much more variability is expected in the *x*‐variable than in the *y*‐variable is challenging” and their suggestion that researchers should “simply report the ratios of variances and interpret their findings in the light of this value” (Price et al., 2014). Price et al. (2014) further stated that if all variables have the same units and are examined on a logarithmic scale that “this approach is valid” (Price et al., 2014). We agree that taking the logarithm of variables prior to the application of criterion (1) makes highly skewed distributions less skewed. However, this transformation does not solve the problem that traits could have different units or do considerably differ in mean values. In our study, body masses of compared amphibians and reptiles comprise different orders of magnitude but both vertebrate groups have similar life history trait values (e.g., amphibians: median of log_10_ body mass = 0.97, reptiles: median of log_10_ body mass = 2.32, Figures 1 and 2). To consider this, we used relative variances of traits in order to apply criterion (1). To do this, we divided raw data on body mass and life history traits by their medians prior to the calculation of variance ratios (Steuer et al., 2007). Without using standardized variances, criterion (1) indicated invariance for all reptilian life history traits (results not shown). This result would strongly contradict previous publications showing that life history traits of reptiles are strongly related to body mass (Dol'nik, 2000; Dunham & Miles, 1985; Hallmann & Griebeler, 2015, 2018; Peters, 1983; Scharf et al., 2014).

Price et al. (2014) suggested inspecting by eye whether life history traits follow a unimodal distribution and then check whether 95% of values are observed within ± 2 *SD* of the mean (criterion 2). In reptiles, except for the trait clutches p.a., criterion (2) was passed by all life history traits, that is, life history traits had statistically confirmed unimodal distributions (Table 2, Figure 2). In contrast, all other criteria (including criterion 1, but also criteria 3, 4, and 5) indicated that almost all reptilian life history traits studied depend on body mass, which in this situation is the strongest evidence on invariance toward body size that we can have under the Price et al. (2014) framework for this trait. Price et al. (2014) did no statistical test on criterion (2), whereas we conducted Hardigan's dip tests on the unimodality of trait values. This introduces the problem that this test (as any other test aiming at deviations from an expected distribution) is very conservative as its null hypothesis is that no deviation exists (to control the type 1 error). Its conservativeness even strengthens when sample sizes are small (e.g., birthweight, egg mass). We, therefore, preferred the test on unimodality followed by a check of whether 95% of trait values are within ± 2 *SD* of the mean over a single test on the normality of data. A further problem with criterion (2) is that it ignores the variance value itself of the trait distribution. A large trait variance contradicts an optimum value (Charnov, 1993; Savage et al., 2006) irrespective of whether the trait distribution is normal or not. Price et al. (2014) already noted that “some traits will have frequency distributions which depart from normality, yet still contain much less variability.” Consistent with both arguments, the application of criteria (1) and (2) revealed inconsistent results for several traits. For example, in all amphibians, criterion (1) rejected invariance for egg mass, whereas egg mass passed criterion (2) or age at maturity passed criterion (1), but criterion (2) was rejected for this trait (Table 2). For all these reasons, we recommend calling a trait only type I invariant toward body size transformation if criteria (1) and (2) indicate invariance.

Identification of type II invariant life history traits. Price et al. (2014) suggested establishing an OLS regression model relating trait values to body mass in order to explore type II invariance. Low slopes (criterion 3) and low coefficients of determination (criterion 4) would then indicate that body mass has low explanatory power and that the trait is not related to body mass, respectively. In our study, we additionally conducted PGLS analysis. The shared evolutionary history implies a covariation in traits across species. Thus, residuals are statistically dependent and OLS assumptions are violated. Using phylogenetically dependent data in OLS analysis could reveal wrong estimates for regression coefficients, causes type 1 error inflation, could lead to wrong standard errors of estimated coefficients, and to wrong confidence and prediction belts of regressions (Felsenstein, 1985; Harvey & Pagel, 1991). In our study, OLS and PGLS analysis on criterion (3) revealed for the majority of analyses conducted consistent results with respect to type II invariance. Exceptions were only observed for the Caudata. The PGLS slope, but not the OLS slope, indicated type II invariance for age at maturity. The OLS slope, but not the PGLS slope, indicated type II for size at maturity (Table 3). We attribute the latter differences to small datasets and/or a strong phylogenetic relatedness of caudatan species as indicated by respective large lambda values (Table 3).

For the majority of OLS regressions, criteria (3) and (4) also revealed consistent results. There were only a few exceptions. The slope was not significant for birthweight in Anura, and for larval period in Caudata, but *R* ^2^ was higher than our threshold (.05) for both traits. For offspring size, we observed the opposite pattern in all amphibians and Anura (Table 3). We explain the first observation by increased confidence intervals of slopes resulting from small sample sizes on birthweight and larval period. The second situation emerged under a large sample size (our sample on offspring size was based on more than 250 species) and a high statistical power for a significant slope close to zero. This demonstrates a further statistical problem with criterion (3), that is, the larger a sample the more unlikely it is that we can statistically confirm no effect of body mass on a trait. We suggest that in such a situation, only the slope value itself should be interpreted in order to check a trait for type II invariance. A slope close to zero indicates almost no impact of body mass on the trait.

To be the most conservative, we decided to assume type II invariance for a trait when OLS slope, PGLS slope, and R^2^ all point to invariance. The results obtained herein for amphibian and reptilian traits show that in the majority of cases, criteria (3) and (4) for OLS analysis and criterion (3) for PGLS analysis reveal similar results especially when sample sizes are large and slope estimates are considerably larger than zero.

Because criterion (5) is so frequently used in the study of type II invariance (Charnov, 1993) and it was used in Price et al. (2014), we also analyzed relationships between pairs of life history traits (not body mass) in order to test whether they scale isometrically. An isometric relationship between two traits ultimately leads to products or ratios of life history traits, which can be dimensionless numbers (Charnov, 1993; Günther & Morgado, 2005; Savage et al., 2006). Some authors warn that “caution is needed when allometric equations are multiplied or divided to make new ones” (Nespolo, 2005). Nee et al. (2005) stated that approaches used so far have “created an illusion of invariants that do not necessarily exist.” They especially criticized that a regression slope or an *R* ^2^ value equaling approximately unity does not imply invariance (Nee et al., 2005).

Our application of the isometric variation criterion (5) identified few invariant trait combinations for amphibians and reptiles (Table 4, Table S2), but for the reasons already listed above (Nee et al., 2005), we think that none of these is a true invariance. Maybe a phylogeny‐informed reduced major axis regression would have uncovered this because confidence intervals of SMA slopes from the standard analysis could potentially be inflated (Warton et al., 2012; Table 4). To cope with statistical shortcomings, Nee et al. (2005) suggested replacing the regressions relating traits against each other by “procedures to compare the relative variation in the proposed invariant across species to variation in other scale‐free, but not necessarily invariant, measures.” The development of such a procedure is out of the scope of our study.

We demonstrated that the five criteria proposed by Price et al. (2014) on type I, II, and I + II invariant traits work for amphibians and reptiles, although we identified some new limitations to their application. Not all criteria have equal power, and the statistical methods have to be adapted to the studied taxa in order to avoid erroneous conclusions and comparisons between taxa. Nevertheless, we think that criteria (1) through (4) from Price et al. (2014) together with our improvements provide a robust decision framework to assess whether a life history trait is invariant or not. We agree that criterion (5) indeed needs further statistical improvement (Nee et al., 2005).

## CONFLICT OF INTEREST

The authors declare that they have no conflict of interest.

## AUTHOR CONTRIBUTIONS

KH wrote the first draft of the manuscript. KH and EMG jointly designed the study, compiled, and analyzed the data, and interpreted the results. KH and EMG equally prepared the final manuscript.

## Data Availability

Amphibian life history data are available online on Dryad Digital Repository (https://doi.org/10.5061/dryad.9cnp5hqdt).
