# Supplementary material for: An identification of invariants in life history traits of amphibians and reptiles
Source: Ecol Evol. 2020 Jan 8;10(3):1233–51. doi: 10.1002/ece3.5978 (PMC7029084; doi:10.1002/ece3.5978)

**Figure S1** Histograms on log_10_-transformed data for each of the amphibian life history traits studied (criterion 1 and 2, type I invariance). A) Anura are in blue and Caudata in red. B) Gymnophiona. Figure 1 in the main text has trait histograms for all amphibians. Information on life history traits of Gymnophiona is sparse, although our final amphibian database (Table S1) covers a total of 6779 species of which 5974 are Anura, 619 are Caudata, and 185 Gymnophiona.

The dashed lines in A) represent the mean value of each trait distribution and the two solid lines represent plus/minus two standard deviations (±2 SD) of the mean.
Sample sizes on Anura: N_body mass_ = 513, N_age at maturity_ = 231, N_birth weight_ = 12, N_offspring size_ = 1121, N_clutch size_ = 1429, N_reproductive output_ = 3902, N_egg mass_ = 12, N_incubation time_ = 46, N_larval period_ = 26, N_maximum longevity_ = 229, N_metamorphosis size_ = 36, N_size at maturity_ = 231;
Sample sizes on Caudata: N_body mass_ = 76, N_age at maturity_ = 162, N_birth weight_ = 7, N_offspring size_ = 191, N_clutch size_= 177, N_reproductive output_ = 484, N_egg mass_ = 7, N_incubation time_ = 39, N_larval period_ = 18, N_maximum longevity_ = 136, N_metamorphosis size_ = 31, N_size at maturity_ = 136.

Sample sizes on Gymnophiona: N_body mass_ = 10, N_age at maturity_ = 6, N_birth weight_ = 0, N_offspring size_ = 21, N_clutch size_ = 23, N_reproductive output_ = 49, N_egg mass_ = 0, N_incubation time_ = 0, N_larval period_ = 0, N_maximum longevity_= 4, N_metamorphosis size_ = 0, N_size at maturity_ = 4.

A)


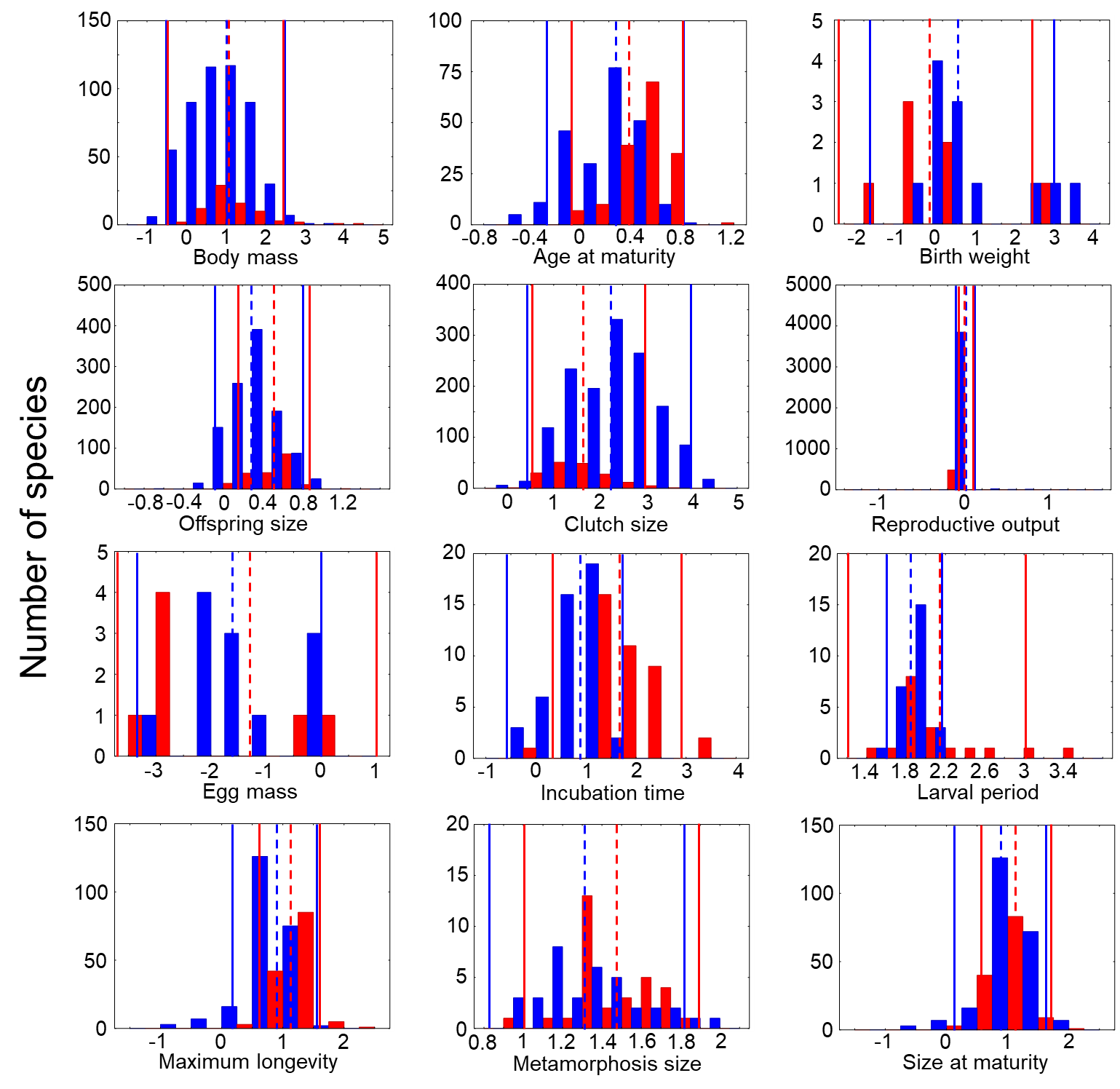


B)


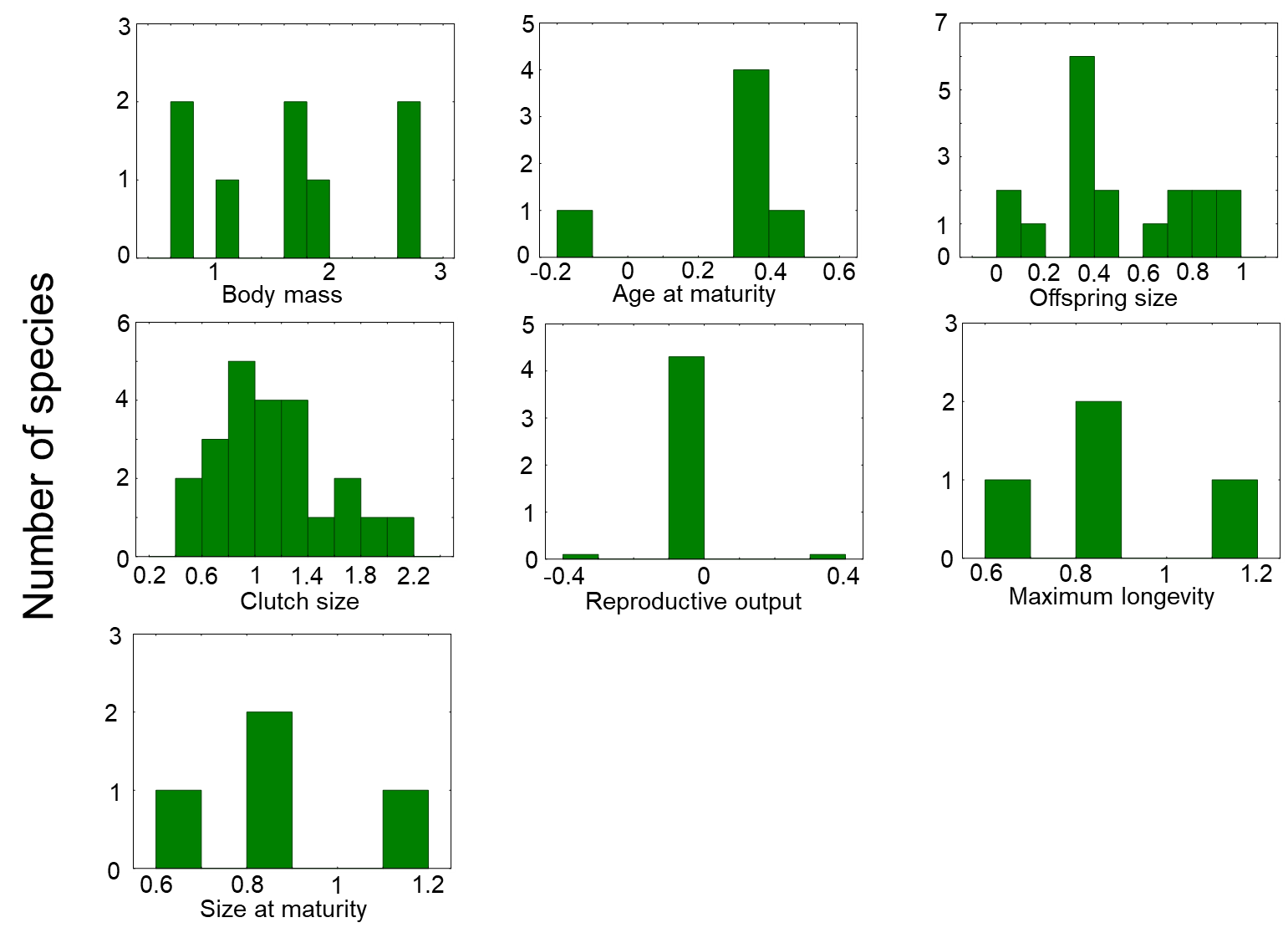

Supplement: Supplementary file 1 [file ECE3-10-1233-s001.docx]
