# Supplementary material for: An identification of invariants in life history traits of amphibians and reptiles
Source: Ecol Evol. 2020 Jan 8;10(3):1233–51. doi: 10.1002/ece3.5978 (PMC7029084; doi:10.1002/ece3.5978)
Supplement: Supplementary file 2 [file ECE3-10-1233-s002.docx]

**Table S1**. Results on invariance of life history traits in the Gymnophiona from Hallmann & Griebeler (2019). For this analysis, we completed our dataset with body sizes of species from the AmphiBIO dataset (Oliveira et al., 2017). Criterion (1) was assessed from body mass estimates as in the main text. As sample sizes for regression analysis (b) are generally small, we only conducted ordinary least squares analysis for exploring criterion (3) and (4) either using body mass or body size as a predictor of the life history trait. Sample sizes: N_body mass_=10, N_body size_=162, N_age at maturity_=6, N_birth weight_=0, N_offspring size_=21, N_clutch size_=23, N_reproductive output_=49, N_egg mass_=0, N_incubation time_=0, N_larval period_=0, N_maximum longevity_=4, N_metamorphosis size_=0, N_size at maturity_=4. For trait distributions see Figure S1.

1. Detailed results on criterion (1) and (2) on type I invariance of life history traits. Variance = variance of the life history trait; body mass var/trait var = ratio of the body mass variance and life history trait variance (both standardized, see main text); Hartigan’s dip test D (p-value) = D and *P*-value of this test on unimodality of the trait distribution; % within ±2 SD = percentage of trait values within plus/minus two standard deviations (SD) of the mean. All traits were log_10_-transformed prior to the application of a criterion. Bold highlights values that are consistent with type I invariance. Infinity = trait variance close to zero.

|  | Age at maturity | Offspring size | Clutch size | Reproductive output | Max. longevity | Size at maturity |
| --- | --- | --- | --- | --- | --- | --- |
| Variance | 0.01 | 0.01 | 0.23 | 0.00 | 0.01 | 0.01 |
| Body mass var/trait var | **26.09** | **39.35** | **2.90** | **Infinity** | **22.43** | **452.59** |
| Hartigan’s dip test D (*P*-value) | 0.17  (**0.05**) | 0.09  (**0.18**) | 0.05  (**0.97**) | 0.01  (**1.00**) | 0.13  (**0.78**) | 0.07  (**0.99**) |
| % within ±2 SD | **100** | 94.44 | **95.65** | **100** | **100** | **100** |

1. Detailed results on criterion (3) and (4) on type II invariance of life history traits. Criteria were only explored under ordinary least squares regression analysis (OLS, Pike et al. 2014) on log_10_-log_10_-transformed data. DF = degrees of freedom; R^2^ = coefficient of determination; Adj R^2^ = adjusted R^2^; p = *P*-value; CI_s_ = 95% confidence interval of slopes; CI_i_ = 95% confidence interval of intercepts. Please note that adjusted R^2^ can drop below zero. This happens if R^2^ is zero or close to zero and usually indicates that the model fits poorly the data. Bold highlights values that are consistent with type II invariance.

| Method |  | DF | R^2^ | Adj R^2^ | p | Slope | CI_S_ | Intercept | CI_i_ |
| --- | --- | --- | --- | --- | --- | --- | --- | --- | --- |
| OLS | Age at maturity ~ body mass | 1 | 0.70 | 0.40 | **0.37** | -0.16 | -1.53, 1.20 | 0.80 | -2.67, 4.28 |
| OLS | Age at maturity ~ body size | 4 | 0.11 | -0.12 | **0.53** | 0.38 | -1.16, 1.92 | -0.73 | -4.89, 3.43 |
| OLS | Offspring size ~ body size | 16 | 0.15 | 0.09 | **0.12** | 0.57 | -0.16, 1.29 | -0.78 | -2.64, 1.08 |
| OLS | Clutch size ~ body mass | 3 | 0.46 | 0.29 | **0.21** | 0.40 | -0.39, 1.19 | 0.56 | -0.87, 1.99 |
| OLS | Clutch size ~ body size | 21 | 0.23 | 0.19 | 0.02 | 0.90 | 0.15, 1.65 | -1.17 | -3.08, 0.74 |
| OLS | Reproductive output ~ body size | 43 | **<0.01** | -0.02 | **0.65** | -0.02 | -0.11, 0.07 | 0.05 | -0.19, 0.30 |
| OLS | Max. longevity ~ body mass | 1 | 0.34 | -0.33 | **0.61** | -0.12 | -2.31 , 2.06 | 1.11 | -4.45 , 6.67 |
| OLS | Max. longevity ~ body size | 2 | 0.18 | -0.23 | **0.57** | -0.85 | -6.32, 4.63 | 3.24 | -12.01, 18.50 |
| OLS | Size at maturity ~ body mass | 1 | 0.99 | 0.98 | **0.06** | 0.05 | -0.01 , 0.10 | 2.35 | 2.24 , 2.46 |
| OLS | Size at maturity ~ body size | 5 | 0.23 | 0.07 | **0.28** | 0.35 | -0.39, 1.09 | 1.49 | -0.48, 3.46 |
