# Supplementary material for: An identification of invariants in life history traits of amphibians and reptiles
Source: Ecol Evol. 2020 Jan 8;10(3):1233–51. doi: 10.1002/ece3.5978 (PMC7029084; doi:10.1002/ece3.5978)
Supplement: Supplementary file 3 [file ECE3-10-1233-s003.docx]

**Table S2**. Results on invariance criterion (5) for life history traits. This criterion is applied to all amphibians, the amphibian orders Anura and Caudata, and the reptiles. For amphibians, results are shown for the dataset compiled for this study (Hallmann & Griebeler (2019). For reptiles, the dataset published by Hallmann & Griebeler (2018) was analyzed. R output of SMA analysis of life history trait combinations is shown. Yellow marks 95% confidence intervals of slopes that include unity and that are consistent with an isometric relation between traits. P-values in red mark slopes consistent with an isometry that are not significant and yellow such that are significant.

1. All amphibians

**Age vs. size at maturity**

Call: sma(formula = Daten$ASM ~ Daten$SSM)

Fit using Standardized Major Axis

------------------------------------------------------------

Coefficients:

elevation slope

estimate -0.2592508 0.6432170

lower limit -0.3240034 0.5846607

upper limit -0.1944983 0.7076381

H0 : variables uncorrelated

R-squared : 0.3301338

P-value : < 2.22e-16

------------------------------------------------------------

**Age at maturity vs. max. longevity**

Call: sma(formula = Daten$ASM ~ Daten$maxL)

Fit using Standardized Major Axis

------------------------------------------------------------

Coefficients:

elevation slope

estimate -0.2885246 0.6905267

lower limit -0.3532676 0.6298965

upper limit -0.2237815 0.7569929

H0 : variables uncorrelated

R-squared : 0.3836001

P-value : < 2.22e-16

------------------------------------------------------------

**Age at maturity vs. egg mass**

Call: sma(formula = Daten$ASM ~ Daten$EM)

Fit using Standardized Major Axis

------------------------------------------------------------

Coefficients:

elevation slope

estimate 0.2261421 -0.10206897

lower limit 0.1218419 -0.16067924

upper limit 0.3304422 -0.06483772

H0 : variables uncorrelated

R-squared : 0.1581788

P-value : 0.091737

------------------------------------------------------------

**Age at maturity vs. clutch size**

Call: sma(formula = Daten$ASM ~ Daten$CS)

Fit using Standardized Major Axis

------------------------------------------------------------

Coefficients:

elevation slope

estimate -0.2989371 0.2672994

lower limit -0.3775733 0.2394832

upper limit -0.2203009 0.2983464

H0 : variables uncorrelated

R-squared : 0.01033398

P-value : 0.070243

------------------------------------------------------------

**Age at maturity vs. birth weight**

Call: sma(formula = Daten$ASM ~ Daten$BW)

Fit using Standardized Major Axis

------------------------------------------------------------

Coefficients:

elevation slope

estimate 0.3723801 0.1166121

lower limit 0.2659309 0.0713483

upper limit 0.4788293 0.1905916

H0 : variables uncorrelated

R-squared : 0.001654865

P-value : 0.86867

------------------------------------------------------------

**Age at maturity vs. offspring size**

Call: sma(formula = Daten$ASM ~ Daten$OS)

Fit using Standardized Major Axis

------------------------------------------------------------

Coefficients:

elevation slope

estimate -0.01191906 1.0345593

lower limit -0.06450214 0.9269825

upper limit 0.04066401 1.1546204

H0 : variables uncorrelated

R-squared : 0.1159309

P-value : 3.6344e-09

------------------------------------------------------------

**Age at maturity vs. reproductive output**

Call: sma(formula = Daten$ASM ~ Daten$RO)

Fit using Standardized Major Axis

------------------------------------------------------------

Coefficients:

elevation slope

estimate 0.3897228 -1.750047

lower limit 0.3574198 -1.929261

upper limit 0.4220257 -1.587481

H0 : variables uncorrelated

R-squared : 0.09502859

P-value : 1.4532e-09

------------------------------------------------------------

**Age at maturity vs. incubation time**

Call: sma(formula = Daten$ASM ~ Daten$IT)

Fit using Standardized Major Axis

------------------------------------------------------------

Coefficients:

elevation slope

estimate -0.06352739 0.3559705

lower limit -0.17777421 0.2876862

upper limit 0.05071942 0.4404626

H0 : variables uncorrelated

R-squared : 0.1416963

P-value : 0.00080406

------------------------------------------------------------

**Age at maturity vs. larval period**

Call: sma(formula = Daten$ASM ~ Daten$LS)

Fit using Standardized Major Axis

------------------------------------------------------------

Coefficients:

elevation slope

estimate -0.7387608 0.5698884

lower limit -1.0499851 0.4363200

upper limit -0.4275365 0.7443453

H0 : variables uncorrelated

R-squared : 0.2468092

P-value : 0.00060296

------------------------------------------------------------

**Age at maturity vs. metamorphosis size**

Call: sma(formula = Daten$ASM ~ Daten$MS)

Fit using Standardized Major Axis

------------------------------------------------------------

Coefficients:

elevation slope

estimate -0.6908249 0.7761483

lower limit -0.9731524 0.6051188

upper limit -0.4084975 0.9955172

H0 : variables uncorrelated

R-squared : 0.01860245

P-value : 0.28253

------------------------------------------------------------

**Size at maturity vs. max. longevity**

Call: sma(formula = Daten$SSM ~ Daten$maxL)

Fit using Standardized Major Axis

------------------------------------------------------------

Coefficients:

elevation slope

estimate -0.038279910 1.055458

lower limit -0.066715555 1.027766

upper limit -0.009844264 1.083896

H0 : variables uncorrelated

R-squared : 0.932898

P-value : < 2.22e-16

------------------------------------------------------------

**Size at maturity vs. egg mass**

Call: sma(formula = Daten$SSM ~ Daten$EM)

Fit using Standardized Major Axis

------------------------------------------------------------

Coefficients:

elevation slope

estimate 1.850773 0.3721211

lower limit 1.409880 0.2199402

upper limit 2.291666 0.6295990

H0 : variables uncorrelated

R-squared : 0.07790464

P-value : 0.29515

------------------------------------------------------------

**Size at maturity vs. clutch size**

Call: sma(formula = Daten$SSM ~ Daten$CS)

Fit using Standardized Major Axis

------------------------------------------------------------

Coefficients:

elevation slope

estimate 0.03345547 0.3865123

lower limit -0.08837672 0.3448930

upper limit 0.15528765 0.4331539

H0 : variables uncorrelated

R-squared : 0.000316062

P-value : 0.75949

------------------------------------------------------------

**Size at maturity vs. birth weight**

Call: sma(formula = Daten$SSM ~ Daten$BW)

Fit using Standardized Major Axis

------------------------------------------------------------

Coefficients:

elevation slope

estimate 1.416556 -0.2666078

lower limit 1.169090 -0.4419874

upper limit 1.664021 -0.1608184

H0 : variables uncorrelated

R-squared : 0.01004298

P-value : 0.69236

------------------------------------------------------------

**Size at maturity vs. offspring size**

Call: sma(formula = Daten$SSM ~ Daten$OS)

Fit using Standardized Major Axis

------------------------------------------------------------

Coefficients:

elevation slope

estimate 0.4617732 1.607331

lower limit 0.3811877 1.429993

upper limit 0.5423586 1.806661

H0 : variables uncorrelated

R-squared : 0.07224973

P-value : 9.4928e-06

------------------------------------------------------------

**Size at maturity vs. reproductive output**

Call: sma(formula = Daten$SSM ~ Daten$RO)

Fit using Standardized Major Axis

------------------------------------------------------------

Coefficients:

elevation slope

estimate 1.0362907 -2.467423

lower limit 0.9851141 -2.738554

upper limit 1.0874673 -2.223136

H0 : variables uncorrelated

R-squared : 0.01579621

P-value : 0.018492

------------------------------------------------------------

**Size at maturity vs. incubation time**

Call: sma(formula = Daten$SSM ~ Daten$IT)

Fit using Standardized Major Axis

------------------------------------------------------------

Coefficients:

elevation slope

estimate 0.6634540 0.4458560

lower limit 0.5084862 0.3555593

upper limit 0.8184218 0.5590840

H0 : variables uncorrelated

R-squared : 0.001925825

P-value : 0.70283

------------------------------------------------------------

**Size at maturity vs. larval period**

Call: sma(formula = Daten$SSM ~ Daten$LS)

Fit using Standardized Major Axis

------------------------------------------------------------

Coefficients:

elevation slope

estimate -0.64589792 0.9656346

lower limit -1.32719967 0.6896042

upper limit 0.03540384 1.3521529

H0 : variables uncorrelated

R-squared : 0.0005069668

P-value : 0.89477

------------------------------------------------------------

**Size at maturity vs. metamorphosis size**

Call: sma(formula = Daten$SSM ~ Daten$MS)

Fit using Standardized Major Axis

------------------------------------------------------------

Coefficients:

elevation slope

estimate 3.257367 -1.459682

lower limit 2.594005 -1.989958

upper limit 3.920729 -1.070711

H0 : variables uncorrelated

R-squared : 0.003296743

P-value : 0.71458

------------------------------------------------------------

**Max. longevity vs. egg mass**

Call: sma(formula = Daten$maxL ~ Daten$EM)

Fit using Standardized Major Axis

------------------------------------------------------------

Coefficients:

elevation slope

estimate 1.627101 0.2876228

lower limit 1.257497 0.1667601

upper limit 1.996705 0.4960832

H0 : variables uncorrelated

R-squared : 0.002535078

P-value : 0.85309

------------------------------------------------------------

**Max. longevity vs. clutch size**

Call: sma(formula = Daten$maxL ~ Daten$CS)

Fit using Standardized Major Axis

------------------------------------------------------------

Coefficients:

elevation slope

estimate 0.07439570 0.3600111

lower limit -0.03976445 0.3211170

upper limit 0.18855586 0.4036160

H0 : variables uncorrelated

R-squared : 8.965691e-05

P-value : 0.87091

------------------------------------------------------------

**Max. longevity vs. birth weight**

Call: sma(formula = Daten$maxL ~ Daten$BW)

Fit using Standardized Major Axis

------------------------------------------------------------

Coefficients:

elevation slope

estimate 1.244245 -0.1810249

lower limit 1.078420 -0.2997167

upper limit 1.410069 -0.1093366

H0 : variables uncorrelated

R-squared : 0.01555088

P-value : 0.622

------------------------------------------------------------

**Max. longevity vs. offspring size**

Call: sma(formula = Daten$maxL ~ Daten$OS)

Fit using Standardized Major Axis

------------------------------------------------------------

Coefficients:

elevation slope

estimate 0.4684369 1.527015

lower limit 0.3927407 1.359086

upper limit 0.5441331 1.715694

H0 : variables uncorrelated

R-squared : 0.08576244

P-value : 1.409e-06

------------------------------------------------------------

**Max. longevity vs. reproductive output**

Call: sma(formula = Daten$maxL ~ Daten$RO)

Fit using Standardized Major Axis

------------------------------------------------------------

Coefficients:

elevation slope

estimate 1.0162253 -2.332595

lower limit 0.9675225 -2.589747

upper limit 1.0649282 -2.100977

H0 : variables uncorrelated

R-squared : 0.01537935

P-value : 0.020482

------------------------------------------------------------

**Max. longevity vs. incubation time**

Call: sma(formula = Daten$maxL ~ Daten$IT)

Fit using Standardized Major Axis

------------------------------------------------------------

Coefficients:

elevation slope

estimate 0.7101274 0.3565497

lower limit 0.5861797 0.2838570

upper limit 0.8340751 0.4478583

H0 : variables uncorrelated

R-squared : 0.01415014

P-value : 0.30608

------------------------------------------------------------

**Max. longevity vs. larval period**

Call: sma(formula = Daten$maxL ~ Daten$LS)

Fit using Standardized Major Axis

------------------------------------------------------------

Coefficients:

elevation slope

estimate -0.1808899 0.6534862

lower limit -0.6482864 0.4647672

upper limit 0.2865065 0.9188347

H0 : variables uncorrelated

R-squared : 0.006346497

P-value : 0.64418

------------------------------------------------------------

**Max. longevity vs. metamorphosis size**

Call: sma(formula = Daten$maxL ~ Daten$MS)

Fit using Standardized Major Axis

------------------------------------------------------------

Coefficients:

elevation slope

estimate 2.627140 -1.0769534

lower limit 2.136623 -1.4688085

upper limit 3.117658 -0.7896391

H0 : variables uncorrelated

R-squared : 0.0005045898

P-value : 0.88631

------------------------------------------------------------

**Egg mass vs. clutch size**

Call: sma(formula = Daten$EM ~ Daten$CS)

Fit using Standardized Major Axis

------------------------------------------------------------

Coefficients:

elevation slope

estimate -4.910954 1.1560242

lower limit -6.659366 0.7090034

upper limit -3.162542 1.8848880

H0 : variables uncorrelated

R-squared : 0.01213973

P-value : 0.65341

------------------------------------------------------------

**Egg mass vs. birth weight**

Call: sma(formula = Daten$EM ~ Daten$BW)

Fit using Standardized Major Axis

------------------------------------------------------------

Coefficients:

elevation slope

estimate -1.47844533 -0.9181396

lower limit -3.03664622 -2.7228045

upper limit 0.07975555 -0.3096000

H0 : variables uncorrelated

R-squared : 0.4884243

P-value : 0.18915

------------------------------------------------------------

**Egg mass vs. offspring size**

Call: sma(formula = Daten$EM ~ Daten$OS)

Fit using Standardized Major Axis

------------------------------------------------------------

Coefficients:

elevation slope

estimate -0.3375698 -4.695561

lower limit -1.2869148 -7.553240

upper limit 0.6117751 -2.919051

H0 : variables uncorrelated

R-squared : 0.1960046

P-value : 0.075131

------------------------------------------------------------

**Egg mass vs. reproductive output**

Call: sma(formula = Daten$EM ~ Daten$RO)

Fit using Standardized Major Axis

------------------------------------------------------------

Coefficients:

elevation slope

estimate -2.229681 5.052941

lower limit -2.916494 3.055359

upper limit -1.542868 8.356536

H0 : variables uncorrelated

R-squared : 0.02032234

P-value : 0.57256

------------------------------------------------------------

**Egg mass vs. incubation time**

Call: sma(formula = Daten$EM ~ Daten$IT)

Fit using Standardized Major Axis

------------------------------------------------------------

Coefficients:

elevation slope

estimate 1.3821050 -2.415616

lower limit -0.5810486 -4.208612

upper limit 3.3452585 -1.386490

H0 : variables uncorrelated

R-squared : 0.04956298

P-value : 0.42515

------------------------------------------------------------

**Egg mass vs. larval period**

Call: sma(formula = Daten$EM ~ Daten$LS)

Fit using Standardized Major Axis

------------------------------------------------------------

Coefficients:

elevation slope

estimate 17.702164 -10.054629

lower limit 4.016219 -19.384162

upper limit 31.388109 -5.215369

H0 : variables uncorrelated

R-squared : 2.498137e-05

P-value : 0.9877

------------------------------------------------------------

**Egg mass vs. metamorphosis size**

Call: sma(formula = Daten$EM ~ Daten$MS)

Fit using Standardized Major Axis

------------------------------------------------------------

Coefficients:

elevation slope

estimate 4.310892 -4.468308

lower limit 1.414120 -6.940482

upper limit 7.207664 -2.876713

H0 : variables uncorrelated

R-squared : 0.3172635

P-value : 0.018553

------------------------------------------------------------

**Clutch size vs. birth weight**

Call: sma(formula = Daten$CS ~ Daten$BW)

Fit using Standardized Major Axis

------------------------------------------------------------

Coefficients:

elevation slope

estimate 2.209657 0.8609454

lower limit 1.456892 0.5283758

upper limit 2.962421 1.4028407

H0 : variables uncorrelated

R-squared : 0.01500645

P-value : 0.61735

------------------------------------------------------------

**Clutch size vs. offspring size**

Call: sma(formula = Daten$CS ~ Daten$OS)

Fit using Standardized Major Axis

------------------------------------------------------------

Coefficients:

elevation slope

estimate 3.265518 -3.375732

lower limit 3.190943 -3.556016

upper limit 3.340094 -3.204588

H0 : variables uncorrelated

R-squared : 0.2188124

P-value : < 2.22e-16

------------------------------------------------------------

**Clutch size vs. reproductive output**

Call: sma(formula = Daten$CS ~ Daten$RO)

Fit using Standardized Major Axis

------------------------------------------------------------

Coefficients:

elevation slope

estimate 2.293641 -8.100611

lower limit 2.231755 -8.531801

upper limit 2.355528 -7.691213

H0 : variables uncorrelated

R-squared : 0.004501075

P-value : 0.011302

------------------------------------------------------------

**Clutch size vs. incubation time**

Call: sma(formula = Daten$CS ~ Daten$IT)

Fit using Standardized Major Axis

------------------------------------------------------------

Coefficients:

elevation slope

estimate 4.391426 -1.504238

lower limit 3.998266 -1.798027

upper limit 4.784585 -1.258453

H0 : variables uncorrelated

R-squared : 0.3334771

P-value : 8.8879e-09

------------------------------------------------------------

**Clutch size vs. larval period**

Call: sma(formula = Daten$CS ~ Daten$IT)

Fit using Standardized Major Axis

------------------------------------------------------------

Coefficients:

elevation slope

estimate 4.391426 -1.504238

lower limit 3.998266 -1.798027

upper limit 4.784585 -1.258453

H0 : variables uncorrelated

R-squared : 0.3334771

P-value : 8.8879e-09

------------------------------------------------------------

**Clutch size vs. metamorphosis size**

Call: sma(formula = Daten$CS ~ Daten$MS)

Fit using Standardized Major Axis

------------------------------------------------------------

Coefficients:

elevation slope

estimate 8.261018 -4.092936

lower limit 6.779755 -5.250124

upper limit 9.742280 -3.190806

H0 : variables uncorrelated

R-squared : 0.001567285

P-value : 0.7542

------------------------------------------------------------

**Birth weight vs. offspring size**

Call: sma(formula = Daten$BW ~ Daten$OS)

Fit using Standardized Major Axis

------------------------------------------------------------

Coefficients:

elevation slope

estimate 1.6189350 -4.503793

lower limit 0.7198959 -7.018753

upper limit 2.5179741 -2.889994

H0 : variables uncorrelated

R-squared : 0.3063137

P-value : 0.021181

------------------------------------------------------------

**Birth weight vs. reproductive output**

Call: sma(formula = Daten$BW ~ Daten$RO)

Fit using Standardized Major Axis

------------------------------------------------------------

Coefficients:

elevation slope

estimate -0.06182651 8.598479

lower limit -0.90863343 5.304005

upper limit 0.78498042 13.939249

H0 : variables uncorrelated

R-squared : 0.03704526

P-value : 0.42986

------------------------------------------------------------

**Birth weight vs. incubation time**

Call: sma(formula = Daten$BW ~ Daten$IT)

Fit using Standardized Major Axis

------------------------------------------------------------

Coefficients:

elevation slope

estimate 2.1108737 -1.4200019

lower limit 0.8506941 -2.4142352

upper limit 3.3710533 -0.8352149

H0 : variables uncorrelated

R-squared : 0.05919975

P-value : 0.36385

------------------------------------------------------------

**Birth weight vs. larval period**

Call: sma(formula = Daten$BW ~ Daten$LS)

Fit using Standardized Major Axis

------------------------------------------------------------

Coefficients:

elevation slope

estimate 11.821791 -5.956716

lower limit 5.049211 -10.379257

upper limit 18.594372 -3.418594

H0 : variables uncorrelated

R-squared : 0.1370833

P-value : 0.19255

------------------------------------------------------------

**Birth weight vs. metamorphosis size**

Call: sma(formula = Daten$BW ~ Daten$MS)

Fit using Standardized Major Axis

------------------------------------------------------------

Coefficients:

elevation slope

estimate 7.260240 -5.279141

lower limit 2.546715 -9.745192

upper limit 11.973765 -2.859803

H0 : variables uncorrelated

R-squared : 0.03432928

P-value : 0.5445

------------------------------------------------------------

**Offspring size vs. reproductive output**

Call: sma(formula = Daten$OS ~ Daten$RO)

Fit using Standardized Major Axis

------------------------------------------------------------

Coefficients:

elevation slope

estimate 0.3524693 -2.364928

lower limit 0.3332617 -2.503548

upper limit 0.3716770 -2.233983

H0 : variables uncorrelated

R-squared : 0.001771603

P-value : 0.14761

------------------------------------------------------------

**Offspring size vs. incubation time**

Call: sma(formula = Daten$OS ~ Daten$IT)

Fit using Standardized Major Axis

------------------------------------------------------------

Coefficients:

elevation slope

estimate -0.16953962 0.4133082

lower limit -0.25903772 0.3516939

upper limit -0.08004152 0.4857170

H0 : variables uncorrelated

R-squared : 0.5305562

P-value : 2.8088e-13

------------------------------------------------------------

**Offspring size vs. larval period**

Call: sma(formula = Daten$OS ~ Daten$LS)

Fit using Standardized Major Axis

------------------------------------------------------------

Coefficients:

elevation slope

estimate -0.9083006 0.5868899

lower limit -1.2655467 0.4344968

upper limit -0.5510545 0.7927326

H0 : variables uncorrelated

R-squared : 0.2089675

P-value : 0.0044498

------------------------------------------------------------

**Offspring size vs. metamorphosis size**

Call: sma(formula = Daten$OS ~ Daten$MS)

Fit using Standardized Major Axis

------------------------------------------------------------

Coefficients:

elevation slope

estimate -1.0910146 1.0362746

lower limit -1.5098505 0.7848839

upper limit -0.6721788 1.3681835

H0 : variables uncorrelated

R-squared : 0.01830984

P-value : 0.33884

------------------------------------------------------------

**Reproductive output vs. incubation time**

Call: sma(formula = Daten$RO ~ Daten$IT)

Fit using Standardized Major Axis

------------------------------------------------------------

Coefficients:

elevation slope

estimate 0.4041248 -0.2994448

lower limit 0.3034904 -0.3720442

upper limit 0.5047591 -0.2410121

H0 : variables uncorrelated

R-squared : 0.008102929

P-value : 0.41547

------------------------------------------------------------

**Reproductive output vs. larval period**

Call: sma(formula = Daten$RO ~ Daten$LS)

Fit using Standardized Major Axis

------------------------------------------------------------

Coefficients:

elevation slope

estimate 1.438034 -0.7114253

lower limit 1.040602 -0.9345510

upper limit 1.835467 -0.5415712

H0 : variables uncorrelated

R-squared : 0.2331675

P-value : 0.0010394

------------------------------------------------------------

**Reproductive output vs. metamorphosis size**

Call: sma(formula = Daten$RO ~ Daten$MS)

Fit using Standardized Major Axis

------------------------------------------------------------

Coefficients:

elevation slope

estimate -0.6393740 0.4700200

lower limit -0.8122431 0.3656072

upper limit -0.4665049 0.6042519

H0 : variables uncorrelated

R-squared : 2.455327e-05

P-value : 0.969

------------------------------------------------------------

**Incubation time vs. larval period**

Call: sma(formula = Daten$IT ~ Daten$LS)

Fit using Standardized Major Axis

------------------------------------------------------------

Coefficients:

elevation slope

estimate -1.6967303 1.492246

lower limit -2.4852394 1.149567

upper limit -0.9082211 1.937076

H0 : variables uncorrelated

R-squared : 0.4449111

P-value : 1.2103e-05

------------------------------------------------------------

**Incubation time vs. metamorphosis size**

Call: sma(formula = Daten$IT ~ Daten$MS)

Fit using Standardized Major Axis

------------------------------------------------------------

Coefficients:

elevation slope

estimate 5.270308 -2.874242

lower limit 3.821679 -4.089836

upper limit 6.718937 -2.019950

H0 : variables uncorrelated

R-squared : 3.980773e-05

P-value : 0.97175

------------------------------------------------------------

**Larval period vs. metamorphosis size**

Call: sma(formula = Daten$LS ~ Daten$MS)

Fit using Standardized Major Axis

------------------------------------------------------------

Coefficients:

elevation slope

estimate 0.2678972 1.2093092

lower limit -0.3487758 0.8549494

upper limit 0.8845703 1.7105440

H0 : variables uncorrelated

R-squared : 0.06736888

P-value : 0.14466

------------------------------------------------------------

1. Anura

**Age vs. size at maturity**

Call: sma(formula = Daten$ASM ~ Daten$SSM)

Fit using Standardized Major Axis

------------------------------------------------------------

Coefficients:

elevation slope

estimate -0.2749203 0.6478949

lower limit -0.3530812 0.5717907

upper limit -0.1967594 0.7341286

H0 : variables uncorrelated

R-squared : 0.29893

P-value : 4.158e-15

------------------------------------------------------------

**Age at maturity vs. max. longevity**

Call: sma(formula = Daten$ASM ~ Daten$maxL)

Fit using Standardized Major Axis

------------------------------------------------------------

Coefficients:

elevation slope

estimate -0.2982426 0.6923687

lower limit -0.3777308 0.6121912

upper limit -0.2187545 0.7830470

H0 : variables uncorrelated

R-squared : 0.3279152

P-value : < 2.22e-16

------------------------------------------------------------

**Age at maturity vs. egg mass**

Call: sma(formula = Daten$ASM ~ Daten$EM)

Fit using Standardized Major Axis

------------------------------------------------------------

Coefficients:

elevation slope

estimate 0.18202070 -0.1274589

lower limit 0.01909488 -0.2356312

upper limit 0.34494652 -0.0689458

H0 : variables uncorrelated

R-squared : 0.1387881

P-value : 0.23302

------------------------------------------------------------

**Age at maturity vs. clutch size**

Call: sma(formula = Daten$ASM ~ Daten$CS)

Fit using Standardized Major Axis

------------------------------------------------------------

Coefficients:

elevation slope

estimate -0.5096929 0.2785354

lower limit -0.6116633 0.2458715

upper limit -0.4077224 0.3155386

H0 : variables uncorrelated

R-squared : 0.1955522

P-value : 4.432e-11

------------------------------------------------------------

**Age at maturity vs. birth weight**

Call: sma(formula = Daten$ASM ~ Daten$BW)

Fit using Standardized Major Axis

------------------------------------------------------------

Coefficients:

elevation slope

estimate 0.4729154 -0.08753081

lower limit 0.3725903 -0.16654212

upper limit 0.5732404 -0.04600423

H0 : variables uncorrelated

R-squared : 0.04504958

P-value : 0.5078

------------------------------------------------------------

**Age at maturity vs. offspring size**

Call: sma(formula = Daten$ASM ~ Daten$OS)

Fit using Standardized Major Axis

------------------------------------------------------------

Coefficients:

elevation slope

estimate -0.03114783 1.236536

lower limit -0.10119196 1.060663

upper limit 0.03889630 1.441572

H0 : variables uncorrelated

R-squared : 0.03298613

P-value : 0.021124

------------------------------------------------------------

**Age at maturity vs. reproductive output**

Call: sma(formula = Daten$ASM ~ Daten$RO)

Fit using Standardized Major Axis

------------------------------------------------------------

Coefficients:

elevation slope

estimate 0.3298700 -1.518674

lower limit 0.2844063 -1.729177

upper limit 0.3753336 -1.333797

H0 : variables uncorrelated

R-squared : 0.06667625

P-value : 0.0001239

------------------------------------------------------------

**Age at maturity vs. incubation time**

Call: sma(formula = Daten$ASM ~ Daten$IT)

Fit using Standardized Major Axis

------------------------------------------------------------

Coefficients:

elevation slope

estimate -0.22211094 0.5621395

lower limit -0.41011999 0.4137688

upper limit -0.03410188 0.7637135

H0 : variables uncorrelated

R-squared : 0.07645912

P-value : 0.080103

------------------------------------------------------------

**Age at maturity vs. larval period**

Call: sma(formula = Daten$ASM ~ Daten$LS)

Fit using Standardized Major Axis

------------------------------------------------------------

Coefficients:

elevation slope

estimate 2.597178 -1.2113175

lower limit 1.649962 -1.8164275

upper limit 3.544394 -0.8077889

H0 : variables uncorrelated

R-squared : 0.02337082

P-value : 0.45592

------------------------------------------------------------

**Age at maturity vs. metamorphosis size**

Call: sma(formula = Daten$ASM ~ Daten$MS)

Fit using Standardized Major Axis

------------------------------------------------------------

Coefficients:

elevation slope

estimate -0.5450613 0.6765928

lower limit -0.8925767 0.4736846

upper limit -0.1975459 0.9664190

H0 : variables uncorrelated

R-squared : 0.01185721

P-value : 0.54637

------------------------------------------------------------

**Size at maturity vs. max. longevity**

Call: sma(formula = Daten$SSM ~ Daten$maxL)

Fit using Standardized Major Axis

------------------------------------------------------------

Coefficients:

elevation slope

estimate -0.027985679 1.045088

lower limit -0.049646788 1.021850

upper limit -0.006324571 1.068855

H0 : variables uncorrelated

R-squared : 0.9704327

P-value : < 2.22e-16

------------------------------------------------------------

**Size at maturity vs. egg mass**

Call: sma(formula = Daten$SSM ~ Daten$EM)

Fit using Standardized Major Axis

------------------------------------------------------------

Coefficients:

elevation slope

estimate 0.3977261 -0.4569928

lower limit -0.3522969 -0.9607399

upper limit 1.1477491 -0.2173767

H0 : variables uncorrelated

R-squared : 0.004839296

P-value : 0.84856

------------------------------------------------------------

**Size at maturity vs. clutch size**

Call: sma(formula = Daten$SSM ~ Daten$CS)

Fit using Standardized Major Axis

------------------------------------------------------------

Coefficients:

elevation slope

estimate -0.2975397 0.4236811

lower limit -0.4748817 0.3687838

upper limit -0.1201977 0.4867505

H0 : variables uncorrelated

R-squared : 0.03843498

P-value : 0.0060185

------------------------------------------------------------

**Size at maturity vs. birth weight**

Call: sma(formula = Daten$SSM ~ Daten$BW)

Fit using Standardized Major Axis

------------------------------------------------------------

Coefficients:

elevation slope

estimate 1.502612 -0.2978780

lower limit 1.145793 -0.5714086

upper limit 1.859432 -0.1552852

H0 : variables uncorrelated

R-squared : 0.01729547

P-value : 0.6837

------------------------------------------------------------

**Size at maturity vs. offspring size**

Call: sma(formula = Daten$SSM ~ Daten$OS)

Fit using Standardized Major Axis

------------------------------------------------------------

Coefficients:

elevation slope

estimate 0.4585062 1.839031

lower limit 0.3592541 1.585020

upper limit 0.5577584 2.133750

H0 : variables uncorrelated

R-squared : 0.03477884

P-value : 0.014596

------------------------------------------------------------

**Size at maturity vs. reproductive output**

Call: sma(formula = Daten$SSM ~ Daten$RO)

Fit using Standardized Major Axis

------------------------------------------------------------

Coefficients:

elevation slope

estimate 0.9757316 -2.219363

lower limit 0.9071405 -2.535382

upper limit 1.0443228 -1.942733

H0 : variables uncorrelated

R-squared : 0.008812108

P-value : 0.16725

------------------------------------------------------------

**Size at maturity vs. incubation time**

Call: sma(formula = Daten$SSM ~ Daten$IT)

Fit using Standardized Major Axis

------------------------------------------------------------

Coefficients:

elevation slope

estimate 1.819637 -0.7345402

lower limit 1.576597 -1.0017352

upper limit 2.062677 -0.5386147

H0 : variables uncorrelated

R-squared : 0.0009773409

P-value : 0.84226

------------------------------------------------------------

**Size at maturity vs. larval period**

Call: sma(formula = Daten$SSM ~ Daten$LS)

Fit using Standardized Major Axis

------------------------------------------------------------

Coefficients:

elevation slope

estimate 5.716248 -2.429805

lower limit 3.717697 -3.726919

upper limit 7.714799 -1.584137

H0 : variables uncorrelated

R-squared : 0.05592361

P-value : 0.27731

------------------------------------------------------------

**Size at maturity vs. metamorphosis size**

Call: sma(formula = Daten$SSM ~ Daten$MS)

Fit using Standardized Major Axis

------------------------------------------------------------

Coefficients:

elevation slope

estimate 3.335761 -1.660963

lower limit 2.385941 -2.512709

upper limit 4.285581 -1.097937

H0 : variables uncorrelated

R-squared : 0.1192618

P-value : 0.10654

------------------------------------------------------------

**Max. longevity vs. egg mass**

Call: sma(formula = Daten$maxL ~ Daten$EM)

Fit using Standardized Major Axis

------------------------------------------------------------

Coefficients:

elevation slope

estimate 0.4558643 -0.4004769

lower limit -0.2060519 -0.8428141

upper limit 1.1177805 -0.1902931

H0 : variables uncorrelated

R-squared : 0.001509734

P-value : 0.91513

------------------------------------------------------------

**Max. longevity vs. clutch size**

Call: sma(formula = Daten$maxL ~ Daten$CS)

Fit using Standardized Major Axis

------------------------------------------------------------

Coefficients:

elevation slope

estimate -0.2588623 0.4015362

lower limit -0.4273871 0.3494517

upper limit -0.0903376 0.4613837

H0 : variables uncorrelated

R-squared : 0.04629185

P-value : 0.0026568

------------------------------------------------------------

**Max. longevity vs. birth weight**

Call: sma(formula = Daten$maxL ~ Daten$BW)

Fit using Standardized Major Axis

------------------------------------------------------------

Coefficients:

elevation slope

estimate 1.279864 -0.2041215

lower limit 1.043212 -0.3893120

upper limit 1.516515 -0.1070236

H0 : variables uncorrelated

R-squared : 0.03691668

P-value : 0.54968

------------------------------------------------------------

**Max. longevity vs. offspring size**

Call: sma(formula = Daten$maxL ~ Daten$OS)

Fit using Standardized Major Axis

------------------------------------------------------------

Coefficients:

elevation slope

estimate 0.4590927 1.756469

lower limit 0.3651644 1.512921

upper limit 0.5530211 2.039224

H0 : variables uncorrelated

R-squared : 0.03831517

P-value : 0.010757

------------------------------------------------------------

**Max. longevity vs. reproductive output**

Call: sma(formula = Daten$maxL ~ Daten$RO)

Fit using Standardized Major Axis

------------------------------------------------------------

Coefficients:

elevation slope

estimate 0.9540468 -2.092961

lower limit 0.8885020 -2.392696

upper limit 1.0195916 -1.830775

H0 : variables uncorrelated

R-squared : 0.007433304

P-value : 0.20691

------------------------------------------------------------

**Max. longevity vs. incubation time**

Call: sma(formula = Daten$maxL ~ Daten$IT)

Fit using Standardized Major Axis

------------------------------------------------------------

Coefficients:

elevation slope

estimate 1.602696 -0.5814341

lower limit 1.410803 -0.7979767

upper limit 1.794588 -0.4236534

H0 : variables uncorrelated

R-squared : 0.01226967

P-value : 0.49054

------------------------------------------------------------

**Max. longevity vs. larval period**

Call: sma(formula = Daten$maxL ~ Daten$LS)

Fit using Standardized Major Axis

------------------------------------------------------------

Coefficients:

elevation slope

estimate 4.275744 -1.718035

lower limit 2.792304 -2.687179

upper limit 5.759185 -1.098417

H0 : variables uncorrelated

R-squared : 0.01732367

P-value : 0.55931

------------------------------------------------------------

**Max. longevity vs. metamorphosis size**

Call: sma(formula = Daten$maxL ~ Daten$MS)

Fit using Standardized Major Axis

------------------------------------------------------------

Coefficients:

elevation slope

estimate 2.811460 -1.3118084

lower limit 2.023725 -2.0217091

upper limit 3.599195 -0.8511814

H0 : variables uncorrelated

R-squared : 0.03346739

P-value : 0.40343

------------------------------------------------------------

**Egg mass vs. clutch size**

Call: sma(formula = Daten$EM ~ Daten$CS)

Fit using Standardized Major Axis

------------------------------------------------------------

Coefficients:

elevation slope

estimate 1.6805453 -1.0738737

lower limit -0.7637183 -2.0504362

upper limit 4.1248090 -0.5624192

H0 : variables uncorrelated

R-squared : 0.03313717

P-value : 0.57123

------------------------------------------------------------

**Egg mass vs. birth weight**

Call: sma(formula = Daten$EM ~ Daten$BW)

Fit using Standardized Major Axis

------------------------------------------------------------

Coefficients:

elevation slope

estimate -1.9416126 -0.5207123

lower limit -3.5682359 -2.4515539

upper limit -0.3149892 -0.1105998

H0 : variables uncorrelated

R-squared : 0.4541321

P-value : 0.32611

------------------------------------------------------------

**Egg mass vs. offspring size**

Call: sma(formula = Daten$EM ~ Daten$OS)

Fit using Standardized Major Axis

------------------------------------------------------------

Coefficients:

elevation slope

estimate 0.03068083 -6.391956

lower limit -1.48193261 -12.773235

upper limit 1.54329427 -3.198650

H0 : variables uncorrelated

R-squared : 0.01347236

P-value : 0.73397

------------------------------------------------------------

**Egg mass vs. reproductive output**

Call: sma(formula = Daten$EM ~ Daten$RO)

Fit using Standardized Major Axis

------------------------------------------------------------

Coefficients:

elevation slope

estimate -2.049386 3.706013

lower limit -2.880867 1.927028

upper limit -1.217906 7.127314

H0 : variables uncorrelated

R-squared : 0.00849213

P-value : 0.77577

------------------------------------------------------------

**Egg mass vs. incubation time**

Call: sma(formula = Daten$EM ~ Daten$IT)

Fit using Standardized Major Axis

------------------------------------------------------------

Coefficients:

elevation slope

estimate 0.8813047 -2.292553

lower limit -1.4494977 -4.825042

upper limit 3.2121072 -1.089275

H0 : variables uncorrelated

R-squared : 0.001310425

P-value : 0.92092

------------------------------------------------------------

**Egg mass vs. larval period**

Call: sma(formula = Daten$EM ~ Daten$LS)

Fit using Standardized Major Axis

------------------------------------------------------------

Coefficients:

elevation slope

estimate 9.028904 -5.701170

lower limit -1.839519 -13.696435

upper limit 19.897328 -2.373124

H0 : variables uncorrelated

R-squared : 0.01173473

P-value : 0.79847

------------------------------------------------------------

**Egg mass vs. metamorphosis size**

Call: sma(formula = Daten$EM ~ Daten$MS)

Fit using Standardized Major Axis

------------------------------------------------------------

Coefficients:

elevation slope

estimate 4.34527788 -4.668001

lower limit 0.01133008 -8.956094

upper limit 8.67922568 -2.433006

H0 : variables uncorrelated

R-squared : 0.1414185

P-value : 0.25436

------------------------------------------------------------

**Clutch size vs. birth weight**

Call: sma(formula = Daten$CS ~ Daten$BW)

Fit using Standardized Major Axis

------------------------------------------------------------

Coefficients:

elevation slope

estimate 3.520207 -0.5413255

lower limit 2.918054 -1.0226692

upper limit 4.122360 -0.2865377

H0 : variables uncorrelated

R-squared : 0.06878614

P-value : 0.41022

------------------------------------------------------------

**Clutch size vs. offspring size**

Call: sma(formula = Daten$CS ~ Daten$OS)

Fit using Standardized Major Axis

------------------------------------------------------------

Coefficients:

elevation slope

estimate 3.277439 -3.421084

lower limit 3.199509 -3.620811

upper limit 3.355368 -3.232374

H0 : variables uncorrelated

R-squared : 0.1915654

P-value : < 2.22e-16

------------------------------------------------------------

**Clutch size vs. reproductive output**

Call: sma(formula = Daten$CS ~ Daten$RO)

Fit using Standardized Major Axis

------------------------------------------------------------

Coefficients:

elevation slope

estimate 2.392772 -7.957918

lower limit 2.327475 -8.410047

upper limit 2.458068 -7.530096

H0 : variables uncorrelated

R-squared : 0.008863024

P-value : 0.00086053

------------------------------------------------------------

**Clutch size vs. incubation time**

Call: sma(formula = Daten$CS ~ Daten$IT)

Fit using Standardized Major Axis

------------------------------------------------------------

Coefficients:

elevation slope

estimate 4.852608 -2.055489

lower limit 4.237290 -2.730423

upper limit 5.467927 -1.547393

H0 : variables uncorrelated

R-squared : 0.102892

P-value : 0.029745

------------------------------------------------------------

**Clutch size vs. larval period**

Call: sma(formula = Daten$CS ~ Daten$LS)

Fit using Standardized Major Axis

------------------------------------------------------------

Coefficients:

elevation slope

estimate 10.492426 -4.015296

lower limit 7.332111 -6.035738

upper limit 13.652742 -2.671190

H0 : variables uncorrelated

R-squared : 0.01100777

P-value : 0.61

------------------------------------------------------------

**Clutch size vs. metamorphosis size**

Call: sma(formula = Daten$CS ~ Daten$MS)

Fit using Standardized Major Axis

------------------------------------------------------------

Coefficients:

elevation slope

estimate -0.4387902 2.680816

lower limit -1.7291873 1.907609

upper limit 0.8516069 3.767426

H0 : variables uncorrelated

R-squared : 0.009457807

P-value : 0.57258

------------------------------------------------------------

**Birth weight vs. offspring size**

Call: sma(formula = Daten$BW ~ Daten$OS)

Fit using Standardized Major Axis

------------------------------------------------------------

Coefficients:

elevation slope

estimate 2.0256136 -7.799319

lower limit 0.9357858 -13.319376

upper limit 3.1154413 -4.566984

H0 : variables uncorrelated

R-squared : 0.3658446

P-value : 0.037196

------------------------------------------------------------

**Birth weight vs. reproductive output**

Call: sma(formula = Daten$BW ~ Daten$RO)

Fit using Standardized Major Axis

------------------------------------------------------------

Coefficients:

elevation slope

estimate 0.116542 6.710821

lower limit -1.031969 3.509117

upper limit 1.265053 12.833746

H0 : variables uncorrelated

R-squared : 0.02777366

P-value : 0.60469

------------------------------------------------------------

**Birth weight vs. incubation time**

Call: sma(formula = Daten$BW ~ Daten$IT)

Fit using Standardized Major Axis

------------------------------------------------------------

Coefficients:

elevation slope

estimate 3.912905 -3.435864

lower limit 1.104699 -6.860634

upper limit 6.721110 -1.720710

H0 : variables uncorrelated

R-squared : 0.01603742

P-value : 0.71061

------------------------------------------------------------

**Birth weight vs. larval period**

Call: sma(formula = Daten$BW ~ Daten$LS)

Fit using Standardized Major Axis

------------------------------------------------------------

Coefficients:

elevation slope

estimate -21.529042 11.77181

lower limit -38.055299 5.87578

upper limit -5.002785 23.58419

H0 : variables uncorrelated

R-squared : 0.005030608

P-value : 0.83583

------------------------------------------------------------

**Birth weight vs. metamorphosis size**

Call: sma(formula = Daten$BW ~ Daten$MS)

Fit using Standardized Major Axis

------------------------------------------------------------

Coefficients:

elevation slope

estimate 6.9960416 -4.849321

lower limit -0.6169925 -12.963520

upper limit 14.6090757 -1.814007

H0 : variables uncorrelated

R-squared : 6.716965e-06

P-value : 0.9956

------------------------------------------------------------

**Offspring size vs. reproductive output**

Call: sma(formula = Daten$OS ~ Daten$RO)

Fit using Standardized Major Axis

------------------------------------------------------------

Coefficients:

elevation slope

estimate 0.3220661 -2.227676

lower limit 0.3016841 -2.370258

upper limit 0.3424480 -2.093670

H0 : variables uncorrelated

R-squared : 0.0001875846

P-value : 0.66516

------------------------------------------------------------

**Offspring size vs. incubation time**

Call: sma(formula = Daten$OS ~ Daten$IT)

Fit using Standardized Major Axis

------------------------------------------------------------

Coefficients:

elevation slope

estimate -0.17770516 0.4312864

lower limit -0.29388838 0.3326187

upper limit -0.06152194 0.5592228

H0 : variables uncorrelated

R-squared : 0.2882692

P-value : 0.00017168

------------------------------------------------------------

**Offspring size vs. larval period**

Call: sma(formula = Daten$OS ~ Daten$LS)

Fit using Standardized Major Axis

------------------------------------------------------------

Coefficients:

elevation slope

estimate -2.063571 1.2054471

lower limit -3.010087 0.8020577

upper limit -1.117054 1.8117183

H0 : variables uncorrelated

R-squared : 0.05736147

P-value : 0.24888

------------------------------------------------------------

**Offspring size vs. metamorphosis size**

Call: sma(formula = Daten$OS ~ Daten$MS)

Fit using Standardized Major Axis

------------------------------------------------------------

Coefficients:

elevation slope

estimate -0.6990953 0.7017347

lower limit -1.0612686 0.4862360

upper limit -0.3369220 1.0127420

H0 : variables uncorrelated

R-squared : 0.1339824

P-value : 0.055409

------------------------------------------------------------

**Reproductive output vs. incubation time**

Call: sma(formula = Daten$RO ~ Daten$IT)

Fit using Standardized Major Axis

------------------------------------------------------------

Coefficients:

elevation slope

estimate -0.3608321 0.4656498

lower limit -0.5040847 0.3484921

upper limit -0.2175795 0.6221942

H0 : variables uncorrelated

R-squared : 0.06433261

P-value : 0.08897

------------------------------------------------------------

**Reproductive output vs. larval period**

Call: sma(formula = Daten$RO ~ Daten$LS)

Fit using Standardized Major Axis

------------------------------------------------------------

Coefficients:

elevation slope

estimate -2.815515 1.553250

lower limit -4.039632 1.032805

upper limit -1.591398 2.335955

H0 : variables uncorrelated

R-squared : 0.008528026

P-value : 0.65366

------------------------------------------------------------

**Reproductive output vs. metamorphosis size**

Call: sma(formula = Daten$RO ~ Daten$MS)

Fit using Standardized Major Axis

------------------------------------------------------------

Coefficients:

elevation slope

estimate -0.7647017 0.5937586

lower limit -1.0704427 0.4151095

upper limit -0.4589606 0.8492922

H0 : variables uncorrelated

R-squared : 0.003737715

P-value : 0.73538

------------------------------------------------------------

**Incubation time vs. larval period**

Call: sma(formula = Daten$IT ~ Daten$LS)

Fit using Standardized Major Axis

------------------------------------------------------------

Coefficients:

elevation slope

estimate -3.061085 2.200826

lower limit -4.550294 1.546726

upper limit -1.571876 3.131542

H0 : variables uncorrelated

R-squared : 0.3368911

P-value : 0.0029439

------------------------------------------------------------

**Incubation time vs. metamorphosis size**

Call: sma(formula = Daten$IT ~ Daten$MS)

Fit using Standardized Major Axis

------------------------------------------------------------

Coefficients:

elevation slope

estimate -0.985598641 1.5150621

lower limit -1.973863276 0.9569954

upper limit 0.002665995 2.3985623

H0 : variables uncorrelated

R-squared : 0.01835621

P-value : 0.55817

------------------------------------------------------------

**Larval period vs. metamorphosis size**

Call: sma(formula = Daten$LS ~ Daten$MS)

Fit using Standardized Major Axis

------------------------------------------------------------

Coefficients:

elevation slope

estimate 1.034250 0.6396252

lower limit 0.575916 0.3861079

upper limit 1.492585 1.0596012

H0 : variables uncorrelated

R-squared : 0.01316962

P-value : 0.65024

------------------------------------------------------------

1. Caudata

**Age vs. size at maturity**

Call: sma(formula = Daten$ASM ~ Daten$SSM)

Fit using Standardized Major Axis

------------------------------------------------------------

Coefficients:

elevation slope

estimate -0.14770984 0.5611933

lower limit -0.26587635 0.4704126

upper limit -0.02954332 0.6694928

H0 : variables uncorrelated

R-squared : 0.1352082

P-value : 7.7546e-05

------------------------------------------------------------

**Age at maturity vs. max. longevity**

Call: sma(formula = Daten$ASM ~ Daten$maxL)

Fit using Standardized Major Axis

------------------------------------------------------------

Coefficients:

elevation slope

estimate -0.20780792 0.6292173

lower limit -0.32855936 0.5326829

upper limit -0.08705649 0.7432459

H0 : variables uncorrelated

R-squared : 0.2304387

P-value : 1.1162e-07

------------------------------------------------------------

**Age at maturity vs. egg mass**

Call: sma(formula = Daten$ASM ~ Daten$EM)

Fit using Standardized Major Axis

------------------------------------------------------------

Coefficients:

elevation slope

estimate 0.27134651 -0.08018155

lower limit 0.06230523 -0.19921947

upper limit 0.48038779 -0.03227135

H0 : variables uncorrelated

R-squared : 0.1799104

P-value : 0.34291

------------------------------------------------------------

**Age at maturity vs. clutch size**

Call: sma(formula = Daten$ASM ~ Daten$CS)

Fit using Standardized Major Axis

------------------------------------------------------------

Coefficients:

elevation slope

estimate -0.06222486 0.3147786

lower limit -0.16906362 0.2618797

upper limit 0.04461391 0.3783629

H0 : variables uncorrelated

R-squared : 0.005520979

P-value : 0.42795

------------------------------------------------------------

**Age at maturity vs. birth weight**

Call: sma(formula = Daten$ASM ~ Daten$BW)

Fit using Standardized Major Axis

------------------------------------------------------------

Coefficients:

elevation slope

estimate 0.423276 0.1626660

lower limit 0.140191 0.0622482

upper limit 0.706361 0.4250764

H0 : variables uncorrelated

R-squared : 0.05885747

P-value : 0.60015

------------------------------------------------------------

**Age at maturity vs. offspring size**

Call: sma(formula = Daten$ASM ~ Daten$OS)

Fit using Standardized Major Axis

------------------------------------------------------------

Coefficients:

elevation slope

estimate 0.008304932 0.916906

lower limit -0.082755074 0.769371

upper limit 0.099364937 1.092732

H0 : variables uncorrelated

R-squared : 0.03200811

P-value : 0.046797

------------------------------------------------------------

**Age at maturity vs. reproductive output**

Call: sma(formula = Daten$ASM ~ Daten$RO)

Fit using Standardized Major Axis

------------------------------------------------------------

Coefficients:

elevation slope

estimate 0.4532112 -2.098349

lower limit 0.4145501 -2.452891

upper limit 0.4918723 -1.795052

H0 : variables uncorrelated

R-squared : 0.04957891

P-value : 0.0056675

------------------------------------------------------------

**Age at maturity vs. incubation time**

Call: sma(formula = Daten$ASM ~ Daten$IT)

Fit using Standardized Major Axis

------------------------------------------------------------

Coefficients:

elevation slope

estimate -0.02910687 0.3077811

lower limit -0.22994304 0.2185951

upper limit 0.17172930 0.4333548

H0 : variables uncorrelated

R-squared : 0.02959423

P-value : 0.32307

------------------------------------------------------------

**Age at maturity vs. larval period**

Call: sma(formula = Daten$ASM ~ Daten$LS)

Fit using Standardized Major Axis

------------------------------------------------------------

Coefficients:

elevation slope

estimate -0.45143876 0.4315377

lower limit -0.81711476 0.2963417

upper limit -0.08576276 0.6284123

H0 : variables uncorrelated

R-squared : 0.4729515

P-value : 0.0016091

------------------------------------------------------------

**Age at maturity vs. metamorphosis size**

Call: sma(formula = Daten$ASM ~ Daten$MS)

Fit using Standardized Major Axis

------------------------------------------------------------

Coefficients:

elevation slope

estimate -0.9205659 0.9268629

lower limit -1.4389873 0.6411356

upper limit -0.4021446 1.3399269

H0 : variables uncorrelated

R-squared : 0.01481497

P-value : 0.51423

------------------------------------------------------------

**Size at maturity vs. max. longevity**

Call: sma(formula = Daten$SSM ~ Daten$maxL)

Fit using Standardized Major Axis

------------------------------------------------------------

Coefficients:

elevation slope

estimate -0.08412888 1.094414

lower limit -0.17969048 1.014290

upper limit 0.01143272 1.180868

H0 : variables uncorrelated

R-squared : 0.7955719

P-value : < 2.22e-16

------------------------------------------------------------

**Size at maturity vs. egg mass**

Call: sma(formula = Daten$SSM ~ Daten$EM)

Fit using Standardized Major Axis

------------------------------------------------------------

Coefficients:

elevation slope

estimate 1.844575 0.2545530

lower limit 1.428589 0.1298634

upper limit 2.260560 0.4989647

H0 : variables uncorrelated

R-squared : 0.7272551

P-value : 0.03091

------------------------------------------------------------

**Size at maturity vs. clutch size**

Call: sma(formula = Daten$SSM ~ Daten$CS)

Fit using Standardized Major Axis

------------------------------------------------------------

Coefficients:

elevation slope

estimate 0.3647306 0.4417618

lower limit 0.2097033 0.3678908

upper limit 0.5197578 0.5304656

H0 : variables uncorrelated

R-squared : 0.1221748

P-value : 0.00027593

------------------------------------------------------------

**Size at maturity vs. birth weight**

Call: sma(formula = Daten$SSM ~ Daten$BW)

Fit using Standardized Major Axis

------------------------------------------------------------

Coefficients:

elevation slope

estimate 1.2840130 -0.22768604

lower limit 0.7275522 -0.70401984

upper limit 1.8404738 -0.07363561

H0 : variables uncorrelated

R-squared : 0.005603285

P-value : 0.88793

------------------------------------------------------------

**Size at maturity vs. offspring size**

Call: sma(formula = Daten$SSM ~ Daten$OS)

Fit using Standardized Major Axis

------------------------------------------------------------

Coefficients:

elevation slope

estimate 0.4256769 1.487054

lower limit 0.2625115 1.209951

upper limit 0.5888424 1.827619

H0 : variables uncorrelated

R-squared : 0.00524591

P-value : 0.49023

------------------------------------------------------------

**Size at maturity vs. reproductive output**

Call: sma(formula = Daten$SSM ~ Daten$RO)

Fit using Standardized Major Axis

------------------------------------------------------------

Coefficients:

elevation slope

estimate 1.148380 2.893546

lower limit 1.079170 2.436369

upper limit 1.217591 3.436510

H0 : variables uncorrelated

R-squared : 0.0001925485

P-value : 0.87404

------------------------------------------------------------

**Size at maturity vs. incubation time**

Call: sma(formula = Daten$SSM ~ Daten$IT)

Fit using Standardized Major Axis

------------------------------------------------------------

Coefficients:

elevation slope

estimate 1.986445 -0.4259924

lower limit 1.709480 -0.6006561

upper limit 2.263410 -0.3021188

H0 : variables uncorrelated

R-squared : 0.02112858

P-value : 0.40476

------------------------------------------------------------

**Size at maturity vs. larval period**

Call: sma(formula = Daten$SSM ~ Daten$LS)

Fit using Standardized Major Axis

------------------------------------------------------------

Coefficients:

elevation slope

estimate 2.912592 -0.6855181

lower limit 1.920073 -1.2381802

upper limit 3.905111 -0.3795369

H0 : variables uncorrelated

R-squared : 0.008553943

P-value : 0.75317

------------------------------------------------------------

**Size at maturity vs. metamorphosis size**

Call: sma(formula = Daten$SSM ~ Daten$MS)

Fit using Standardized Major Axis

------------------------------------------------------------

Coefficients:

elevation slope

estimate -0.9710037 1.4821343

lower limit -2.0955033 0.9215262

upper limit 0.1534959 2.3837868

H0 : variables uncorrelated

R-squared : 0.007644933

P-value : 0.71396

------------------------------------------------------------

**Max. longevity vs. egg mass**

Call: sma(formula = Daten$maxL ~ Daten$EM)

Fit using Standardized Major Axis

------------------------------------------------------------

Coefficients:

elevation slope

estimate 1.486620 0.14604845

lower limit 1.015098 0.04890038

upper limit 1.958141 0.43619600

H0 : variables uncorrelated

R-squared : 0.08775162

P-value : 0.56865

------------------------------------------------------------

**Max. longevity vs. clutch size**

Call: sma(formula = Daten$maxL ~ Daten$CS)

Fit using Standardized Major Axis

------------------------------------------------------------

Coefficients:

elevation slope

estimate 0.4291838 0.3881841

lower limit 0.2896027 0.3220622

upper limit 0.5687649 0.4678813

H0 : variables uncorrelated

R-squared : 0.08540048

P-value : 0.0026108

------------------------------------------------------------

**Max. longevity vs. birth weight**

Call: sma(formula = Daten$maxL ~ Daten$BW)

Fit using Standardized Major Axis

------------------------------------------------------------

Coefficients:

elevation slope

estimate 1.2075934 0.14722725

lower limit 0.8540169 0.04772712

upper limit 1.5611699 0.45416243

H0 : variables uncorrelated

R-squared : 0.01138256

P-value : 0.84057

------------------------------------------------------------

**Max. longevity vs. offspring size**

Call: sma(formula = Daten$maxL ~ Daten$OS)

Fit using Standardized Major Axis

------------------------------------------------------------

Coefficients:

elevation slope

estimate 0.4618544 1.372367

lower limit 0.3122470 1.117292

upper limit 0.6114617 1.685675

H0 : variables uncorrelated

R-squared : 0.01099452

P-value : 0.31718

------------------------------------------------------------

**Max. longevity vs. reproductive output**

Call: sma(formula = Daten$maxL ~ Daten$RO)

Fit using Standardized Major Axis

------------------------------------------------------------

Coefficients:

elevation slope

estimate 1.128634 2.685022

lower limit 1.064308 2.260777

upper limit 1.192960 3.188880

H0 : variables uncorrelated

R-squared : 0.0001144927

P-value : 0.90271

------------------------------------------------------------

**Max. longevity vs. incubation time**

Call: sma(formula = Daten$maxL ~ Daten$IT)

Fit using Standardized Major Axis

------------------------------------------------------------

Coefficients:

elevation slope

estimate 1.789472 -0.3412129

lower limit 1.562654 -0.4828036

upper limit 2.016291 -0.2411462

H0 : variables uncorrelated

R-squared : 0.0002724117

P-value : 0.92503

------------------------------------------------------------

**Max. longevity vs. larval period**

Call: sma(formula = Daten$maxL ~ Daten$LS)

Fit using Standardized Major Axis

------------------------------------------------------------

Coefficients:

elevation slope

estimate 0.2026692 0.4434891

lower limit -0.4430496 0.2450810

upper limit 0.8483879 0.8025205

H0 : variables uncorrelated

R-squared : 0.001585228

P-value : 0.8925

------------------------------------------------------------

**Max. longevity vs. metamorphosis size**

Call: sma(formula = Daten$maxL ~ Daten$MS)

Fit using Standardized Major Axis

------------------------------------------------------------

Coefficients:

elevation slope

estimate -0.3732705 1.0049645

lower limit -1.1379879 0.6241847

upper limit 0.3914470 1.6180364

H0 : variables uncorrelated

R-squared : 0.00291013

P-value : 0.82129

------------------------------------------------------------

**Egg mass vs. clutch size**

Call: sma(formula = Daten$EM ~ Daten$CS)

Fit using Standardized Major Axis

------------------------------------------------------------

Coefficients:

elevation slope

estimate -6.076377 2.0659902

lower limit -10.864013 0.8102626

upper limit -1.288741 5.2678171

H0 : variables uncorrelated

R-squared : 0.1193893

P-value : 0.44779

------------------------------------------------------------

**Egg mass vs. birth weight**

Call: sma(formula = Daten$EM ~ Daten$BW)

**Egg mass vs. offspring size**

Call: sma(formula = Daten$EM ~ Daten$OS)

Fit using Standardized Major Axis

------------------------------------------------------------

Coefficients:

elevation slope

estimate -0.5132143 -4.007555

lower limit -3.0203512 -10.792358

upper limit 1.9939226 -1.488136

H0 : variables uncorrelated

R-squared : 0.3007653

P-value : 0.25984

------------------------------------------------------------

**Egg mass vs. incubation time**

Call: sma(formula = Daten$EM ~ Daten$IT)

Fit using Standardized Major Axis

------------------------------------------------------------

Coefficients:

elevation slope

estimate 2.857497 -2.9139167

lower limit -4.804743 -10.2638456

upper limit 10.519737 -0.8272641

H0 : variables uncorrelated

R-squared : 0.2233699

P-value : 0.42146

------------------------------------------------------------

**Egg mass vs. larval period**

Call: sma(formula = Daten$EM ~ Daten$LS)

**Egg mass vs. metamorphosis size**

Call: sma(formula = Daten$EM ~ Daten$MS)

Fit using Standardized Major Axis

------------------------------------------------------------

Coefficients:

elevation slope

estimate 8.477819 -6.845018

lower limit -1.367905 -15.450055

upper limit 18.323544 -3.032628

H0 : variables uncorrelated

R-squared : 0.5730905

P-value : 0.081382

------------------------------------------------------------

**Clutch size vs. birth weight**

Call: sma(formula = Daten$CS ~ Daten$BW)

Fit using Standardized Major Axis

------------------------------------------------------------

Coefficients:

elevation slope

estimate 1.2894909 -0.4909094

lower limit 0.3453199 -1.3096112

upper limit 2.2336620 -0.1840180

H0 : variables uncorrelated

R-squared : 0.005494243

P-value : 0.87451

------------------------------------------------------------

**Clutch size vs. offspring size**

Call: sma(formula = Daten$CS ~ Daten$OS)

Fit using Standardized Major Axis

------------------------------------------------------------

Coefficients:

elevation slope

estimate 3.093122 -3.011708

lower limit 2.824329 -3.528136

upper limit 3.361915 -2.570871

H0 : variables uncorrelated

R-squared : 0.08222474

P-value : 0.00049313

------------------------------------------------------------

**Clutch size vs. reproductive output**

Call: sma(formula = Daten$CS ~ Daten$RO)

Fit using Standardized Major Axis

------------------------------------------------------------

Coefficients:

elevation slope

estimate 1.571106 -7.224994

lower limit 1.436419 -8.389787

upper limit 1.705794 -6.221915

H0 : variables uncorrelated

R-squared : 0.000496147

P-value : 0.76984

------------------------------------------------------------

**Clutch size vs. incubation time**

Call: sma(formula = Daten$CS ~ Daten$IT)

Fit using Standardized Major Axis

------------------------------------------------------------

Coefficients:

elevation slope

estimate 3.950288 -1.2154929

lower limit 3.260691 -1.6458539

upper limit 4.639885 -0.8976635

H0 : variables uncorrelated

R-squared : 0.1709405

P-value : 0.0098763

------------------------------------------------------------

**Clutch size vs. larval period**

Call: sma(formula = Daten$CS ~ Daten$LS)

Fit using Standardized Major Axis

------------------------------------------------------------

Coefficients:

elevation slope

estimate 4.897396 -1.3372872

lower limit 3.408545 -2.1630123

upper limit 6.386246 -0.8267808

H0 : variables uncorrelated

R-squared : 0.175871

P-value : 0.0938

------------------------------------------------------------

**Clutch size vs. metamorphosis size**

Call: sma(formula = Daten$CS ~ Daten$MS)

Fit using Standardized Major Axis

------------------------------------------------------------

Coefficients:

elevation slope

estimate -3.162211 3.345718

lower limit -4.982217 2.338892

upper limit -1.342205 4.785953

H0 : variables uncorrelated

R-squared : 0.1423073

P-value : 0.043659

------------------------------------------------------------

**Birth weight vs. offspring size**

Call: sma(formula = Daten$BW ~ Daten$OS)

Fit using Standardized Major Axis

------------------------------------------------------------

Coefficients:

elevation slope

estimate -2.2294244 2.476964

lower limit -5.1270447 0.669856

upper limit 0.6681959 9.159207

H0 : variables uncorrelated

R-squared : 0.1301413

P-value : 0.55084

------------------------------------------------------------

**Birth weight vs. reproductive output**

Call: sma(formula = Daten$BW ~ Daten$OS)

Fit using Standardized Major Axis

------------------------------------------------------------

Coefficients:

elevation slope

estimate -2.2294244 2.476964

lower limit -5.1270447 0.669856

upper limit 0.6681959 9.159207

H0 : variables uncorrelated

R-squared : 0.1301413

P-value : 0.55084

------------------------------------------------------------

**Birth weight vs. incubation time**

Call: sma(formula = Daten$BW ~ Daten$IT)

Fit using Standardized Major Axis

------------------------------------------------------------

Coefficients:

elevation slope

estimate -1.8736148 0.7239781

lower limit -3.9640536 0.2456447

upper limit 0.2168239 2.1337494

H0 : variables uncorrelated

R-squared : 0.4963366

P-value : 0.18403

------------------------------------------------------------

**Birth weight vs. larval period**

Call: sma(formula = Daten$BW ~ Daten$LS)

Fit using Standardized Major Axis

------------------------------------------------------------

Coefficients:

elevation slope

estimate 12.89563 -5.9037818

lower limit -109.38766 -111.3819109

upper limit 135.17892 -0.3129291

H0 : variables uncorrelated

R-squared : 0.4519334

P-value : 0.53065

------------------------------------------------------------

**Birth weight vs. metamorphosis size**

Call: sma(formula = Daten$BW ~ Daten$MS)

Fit using Standardized Major Axis

------------------------------------------------------------

Coefficients:

elevation slope

estimate 6.871137 -5.254305

lower limit -2.504954 -15.421954

upper limit 16.247228 -1.790157

H0 : variables uncorrelated

R-squared : 0.1268325

P-value : 0.48838

------------------------------------------------------------

**Offspring size vs. reproductive output**

Call: sma(formula = Daten$OS ~ Daten$RO)

Fit using Standardized Major Axis

------------------------------------------------------------

Coefficients:

elevation slope

estimate 0.5044823 -2.182993

lower limit 0.4644239 -2.525436

upper limit 0.5445406 -1.886983

H0 : variables uncorrelated

R-squared : 0.0002904133

P-value : 0.8184

------------------------------------------------------------

**Offspring size vs. incubation time**

Call: sma(formula = Daten$OS ~ Daten$IT)

Fit using Standardized Major Axis

------------------------------------------------------------

Coefficients:

elevation slope

estimate -0.20144431 0.4254595

lower limit -0.41569525 0.3178619

upper limit 0.01280662 0.5694793

H0 : variables uncorrelated

R-squared : 0.4392291

P-value : 8.9542e-05

------------------------------------------------------------

**Offspring size vs. larval period**

Call: sma(formula = Daten$OS ~ Daten$LS)

Fit using Standardized Major Axis

------------------------------------------------------------

Coefficients:

elevation slope

estimate -0.44707725 0.3731104

lower limit -0.95189338 0.2094833

upper limit 0.05773888 0.6645464

H0 : variables uncorrelated

R-squared : 0.2509273

P-value : 0.097135

------------------------------------------------------------

**Offspring size vs. metamorphosis size**

Call: sma(formula = Daten$OS ~ Daten$MS)

Fit using Standardized Major Axis

------------------------------------------------------------

Coefficients:

elevation slope

estimate 2.275188 -1.2116594

lower limit 1.527291 -1.8135340

upper limit 3.023085 -0.8095346

H0 : variables uncorrelated

R-squared : 0.1219807

P-value : 0.094363

------------------------------------------------------------

**Reproductive output vs. incubation time**

Call: sma(formula = Daten$RO ~ Daten$IT)

Fit using Standardized Major Axis

------------------------------------------------------------

Coefficients:

elevation slope

estimate 0.4941292 -0.3032421

lower limit 0.3024279 -0.4217446

upper limit 0.6858305 -0.2180367

H0 : variables uncorrelated

R-squared : 0.01257677

P-value : 0.50264

------------------------------------------------------------

**Reproductive output vs. larval period**

Call: sma(formula = Daten$RO ~ Daten$LS)

Fit using Standardized Major Axis

------------------------------------------------------------

Coefficients:

elevation slope

estimate 1.1171811 -0.5481167

lower limit 0.6202966 -0.8163359

upper limit 1.6140656 -0.3680248

H0 : variables uncorrelated

R-squared : 0.4478029

P-value : 0.0033058

------------------------------------------------------------

**Reproductive output vs. metamorphosis size**

Call: sma(formula = Daten$RO ~ Daten$MS)

**Incubation time vs. larval period**

Call: sma(formula = Daten$IT ~ Daten$LS)

Fit using Standardized Major Axis

------------------------------------------------------------

Coefficients:

elevation slope

estimate -1.0429914 1.2288302

lower limit -2.6960285 0.6967885

upper limit 0.6100456 2.1671190

H0 : variables uncorrelated

R-squared : 0.3705182

P-value : 0.046875

------------------------------------------------------------

**Incubation time vs. metamorphosis size**

Call: sma(formula = Daten$IT ~ Daten$MS)

Fit using Standardized Major Axis

------------------------------------------------------------

Coefficients:

elevation slope

estimate 6.603340 -3.371212

lower limit 3.669076 -6.001522

upper limit 9.537605 -1.893698

H0 : variables uncorrelated

R-squared : 0.1571508

P-value : 0.17991

------------------------------------------------------------

**Larval period vs. metamorphosis size**

Call: sma(formula = Daten$LS ~ Daten$MS)

Fit using Standardized Major Axis

------------------------------------------------------------

Coefficients:

elevation slope

estimate -0.6319019 1.795384

lower limit -2.2509157 1.027365

upper limit 0.9871119 3.137545

H0 : variables uncorrelated

R-squared : 0.03805096

P-value : 0.486

------------------------------------------------------------

1. Reptiles from Hallmann & Griebeler (2018)

**Size_at_maturity vs. birth_weight**

Call: sma(formula = log10(trait2) ~ log10(trait3))

Fit using Standardized Major Axis

------------------------------------------------------------

Coefficients:

elevation slope

estimate 0.8362210 0.7133150

lower limit 0.5458825 0.5172112

upper limit 1.1265595 0.9837727

H0 : variables uncorrelated

R-squared : 0.2029167

P-value : 0.008522

**Size_at_maturity vs. clutch_size**

Call: sma(formula = log10(trait2) ~ log10(trait3))

Fit using Standardized Major Axis

------------------------------------------------------------

Coefficients:

elevation slope

estimate 0.1333367 1.278445

lower limit -0.1818331 1.050194

upper limit 0.4485065 1.556305

H0 : variables uncorrelated

R-squared : 0.5042198

P-value : 2.6113e-09

**Size_at_maturity vs. clutches_pa**

Call: sma(formula = log10(trait2) ~ log10(trait3))

Fit using Standardized Major Axis

------------------------------------------------------------

Coefficients:

elevation slope

estimate 1.826589 -1.828687

lower limit 1.629870 -2.432045

upper limit 2.023307 -1.375014

H0 : variables uncorrelated

R-squared : 0.1168676

P-value : 0.021536

**Size_at_maturity vs. egg_weight**

Call: sma(formula = log10(trait2) ~ log10(trait3))

Fit using Standardized Major Axis

------------------------------------------------------------

Coefficients:

elevation slope

estimate 0.7820691 0.7664760

lower limit 0.6049032 0.6552937

upper limit 0.9592349 0.8965223

H0 : variables uncorrelated

R-squared : 0.8090161

P-value : 4.8406e-13

**Size_at_maturity vs. age_at_maturity**

Call: sma(formula = log10(trait2) ~ log10(trait3))

Fit using Standardized Major Axis

------------------------------------------------------------

Coefficients:

elevation slope

estimate -3.562195 1.564586

lower limit -4.977810 1.199449

upper limit -2.146579 2.040880

H0 : variables uncorrelated

R-squared : 0.2543239

P-value : 0.00048221

**Size_at_maturity vs. incubation_time**

Call: sma(formula = log10(trait2) ~ log10(trait3))

Fit using Standardized Major Axis

------------------------------------------------------------

Coefficients:

elevation slope

estimate 8.458976 -3.632783

lower limit 6.504591 -4.806492

upper limit 10.413360 -2.745685

H0 : variables uncorrelated

R-squared : 0.002904623

P-value : 0.70434

**Size_at_maturity vs. max_longevity**

Call: sma(formula = log10(trait2) ~ log10(trait3))

Fit using Standardized Major Axis

------------------------------------------------------------

Coefficients:

elevation slope

estimate -1.0081589 1.865472

lower limit -1.9121989 1.356792

upper limit -0.1041189 2.564863

H0 : variables uncorrelated

R-squared : 0.02783215

P-value : 0.30353

**Birth_weight vs. clutch_size**

Call: sma(formula = log10(trait2) ~ log10(trait3))

Fit using Standardized Major Axis

------------------------------------------------------------

Coefficients:

elevation slope

estimate -0.6530360 1.550786

lower limit -0.9807414 1.274589

upper limit -0.3253306 1.886833

H0 : variables uncorrelated

R-squared : 0.2737225

P-value : 1.2415e-06

**Birth_weight vs. clutches_pa**

Call: sma(formula = log10(trait2) ~ log10(trait3))

Fit using Standardized Major Axis

------------------------------------------------------------

Coefficients:

elevation slope

estimate 1.2096402 -2.227478

lower limit 0.9281371 -2.852570

upper limit 1.4911434 -1.739364

H0 : variables uncorrelated

R-squared : 0.06367572

P-value : 0.047856

**Birth_weight vs egg_weight**

Call: sma(formula = log10(trait2) ~ log10(trait3))

Fit using Standardized Major Axis

------------------------------------------------------------

Coefficients:

elevation slope

estimate -0.1332099 1.0605269

lower limit -0.4264361 0.8403363

upper limit 0.1600163 1.3384133

H0 : variables uncorrelated

R-squared : 0.6033719

P-value : 1.7246e-07

**Birth_weight vs. age_at_maturity**

Call: sma(formula = log10(trait2) ~ log10(trait3))

Fit using Standardized Major Axis

------------------------------------------------------------

Coefficients:

elevation slope

estimate -4.924594 1.817940

lower limit -6.401763 1.413693

upper limit -3.447425 2.337783

H0 : variables uncorrelated

R-squared : 0.3674356

P-value : 2.0959e-05

**Birth_weight vs. incubation_time**

Call: sma(formula = log10(trait2) ~ log10(trait3))

Fit using Standardized Major Axis

------------------------------------------------------------

Coefficients:

elevation slope

estimate -6.410153 3.696508

lower limit -8.054220 2.951890

upper limit -4.766087 4.628957

H0 : variables uncorrelated

R-squared : 0.09444481

P-value : 0.0086414

**Birth_weight ~ max_longevity**

Call: sma(formula = log10(trait2) ~ log10(trait3))

Fit using Standardized Major Axis

------------------------------------------------------------

Coefficients:

elevation slope

estimate -2.380411 2.336157

lower limit -3.199397 1.834331

upper limit -1.561424 2.975269

H0 : variables uncorrelated

R-squared : 0.2764849

P-value : 7.4168e-05

**Clutch_size vs. clutches_pa**

Call: sma(formula = log10(trait2) ~ log10(trait3))

Fit using Standardized Major Axis

------------------------------------------------------------

Coefficients:

elevation slope

estimate 1.181526 -1.603597

lower limit 1.056723 -1.902113

upper limit 1.306328 -1.351931

H0 : variables uncorrelated

R-squared : 0.02257125

P-value : 0.085532

**Clutch_size vs. egg_weight**

Call: sma(formula = log10(trait2) ~ log10(trait3))

Fit using Standardized Major Axis

------------------------------------------------------------

Coefficients:

elevation slope

estimate 0.4847610 0.5814189

lower limit 0.3535384 0.4963087

upper limit 0.6159836 0.6811244

H0 : variables uncorrelated

R-squared : 0.6212078

P-value : 2.8866e-14

**Clutch_size vs. age_at_maturity**

Call: sma(formula = log10(trait2) ~ log10(trait3))

Fit using Standardized Major Axis

------------------------------------------------------------

Coefficients:

elevation slope

estimate -3.020405 1.280844

lower limit -3.650167 1.097406

upper limit -2.390643 1.494944

H0 : variables uncorrelated

R-squared : 0.3505551

P-value : 1.491e-11

**Clutch_size vs. incubation_time**

Call: sma(formula = log10(trait2) ~ log10(trait3))

Fit using Standardized Major Axis

------------------------------------------------------------

Coefficients:

elevation slope

estimate 5.622865 -2.454473

lower limit 4.903192 -2.856552

upper limit 6.342539 -2.108989

H0 : variables uncorrelated

R-squared : 0.0003406935

P-value : 0.81118

**Clutch_size vs. max_longevity**

Call: sma(formula = log10(trait2) ~ log10(trait3))

Fit using Standardized Major Axis

------------------------------------------------------------

Coefficients:

elevation slope

estimate -0.8714328 1.413839

lower limit -1.1834461 1.204908

upper limit -0.5594196 1.658999

H0 : variables uncorrelated

R-squared : 0.1098922

P-value : 7.5884e-05

**Clutches_pa vs. egg_weight**

Call: sma(formula = log10(trait2) ~ log10(trait3))

Fit using Standardized Major Axis

------------------------------------------------------------

Coefficients:

elevation slope

estimate 0.6103383 -0.4574377

lower limit 0.4688223 -0.6045466

upper limit 0.7518542 -0.3461260

H0 : variables uncorrelated

R-squared : 0.2394356

P-value : 0.0011674

**Clutches_pa vs. age_at_maturity**

Call: sma(formula = log10(trait2) ~ log10(trait3))

Fit using Standardized Major Axis

------------------------------------------------------------

Coefficients:

elevation slope

estimate 2.275059 -0.6749577

lower limit 1.798229 -0.8454472

upper limit 2.751889 -0.5388484

H0 : variables uncorrelated

R-squared : 0.01166245

P-value : 0.34664

**Clutches_pa vs. incubation_time**

Call: sma(formula = log10(trait2) ~ log10(trait3))

Fit using Standardized Major Axis

------------------------------------------------------------

Coefficients:

elevation slope

estimate 2.978559 -1.434495

lower limit 2.460678 -1.732076

upper limit 3.496441 -1.188041

H0 : variables uncorrelated

R-squared : 0.002233383

P-value : 0.62234

**Clutches_pa vs. max_longevity**

Call: sma(formula = log10(trait2) ~ log10(trait3))

Fit using Standardized Major Axis

------------------------------------------------------------

Coefficients:

elevation slope

estimate 1.1589698 -0.7585161

lower limit 0.9188256 -0.9605560

upper limit 1.3991140 -0.5989725

H0 : variables uncorrelated

R-squared : 0.0002646585

P-value : 0.89211

**Egg_weight vs. age_at_maturity**

Call: sma(formula = log10(trait2) ~ log10(trait3))

Fit using Standardized Major Axis

------------------------------------------------------------

Coefficients:

elevation slope

estimate -5.999069 2.167120

lower limit -7.590293 1.748024

upper limit -4.407845 2.686695

H0 : variables uncorrelated

R-squared : 0.5407006

P-value : 2.919e-08

**Egg_weight vs. incubation_time**

Call: sma(formula = log10(trait2) ~ log10(trait3))

Fit using Standardized Major Axis

------------------------------------------------------------

Coefficients:

elevation slope

estimate -10.648604 6.306428

lower limit -13.670693 4.893729

upper limit -7.626515 8.126939

H0 : variables uncorrelated

R-squared : 0.01462257

P-value : 0.34916

**Egg_weight vs. max_longevity**

Call: sma(formula = log10(trait2) ~ log10(trait3))

Fit using Standardized Major Axis

------------------------------------------------------------

Coefficients:

elevation slope

estimate -2.442019 2.678825

lower limit -3.425763 2.080594

upper limit -1.458275 3.449066

H0 : variables uncorrelated

R-squared : 0.3102176

P-value : 7.0759e-05

**Age_at_maturity vs. incubation_time**

Call: sma(formula = log10(trait2) ~ log10(trait3))

Fit using Standardized Major Axis

------------------------------------------------------------

Coefficients:

elevation slope

estimate -1.0573580 2.233140

lower limit -1.8501545 1.854906

upper limit -0.2645615 2.688499

H0 : variables uncorrelated

R-squared : 0.1603297

P-value : 4.8267e-05

**Age_at_maturity vs. max_longevity**

Call: sma(formula = log10(trait2) ~ log10(trait3))

Fit using Standardized Major Axis

------------------------------------------------------------

Coefficients:

elevation slope

estimate 1.741658 1.0612832

lower limit 1.523188 0.9134007

upper limit 1.960128 1.2331084

H0 : variables uncorrelated

R-squared : 0.4767595

P-value : 1.9096e-14

**Incubation_time vs. max_longevity**

Call: sma(formula = log10(trait2) ~ log10(trait3))

Fit using Standardized Major Axis

------------------------------------------------------------

Coefficients:

elevation slope

estimate 1.196253 0.5465494

lower limit 1.065610 0.4603827

upper limit 1.326896 0.6488434

H0 : variables uncorrelated

R-squared : 0.09007795

P-value : 0.00078311
